# Is the diet cyclic phase‐dependent in boreal vole populations?

**DOI:** 10.1002/ece3.11227

**Published:** 2024-04-17

**Authors:** Magne Neby, Rolf A. Ims, Stefaniya Kamenova, Olivier Devineau, Eeva M. Soininen

**Affiliations:** ^1^ Department of Applied Ecology Inland Norway University of Applied Sciences Koppang Norway; ^2^ Department of Agricultural Sciences Inland Norway University of Applied Sciences Hamar Norway; ^3^ Department of Arctic and Marine Biology UiT – The Arctic University of Norway Tromsø Norway; ^4^ Department of Biosciences, Centre for Ecological and Evolutionary Synthesis University of Oslo Oslo Norway; ^5^ Faculty of Environmental Sciences and Natural Resource Management Norwegian University of Life Sciences Ås Norway; ^6^ National Museum of Natural History Bulgarian Academy of Sciences Sofia Bulgaria

**Keywords:** density dependence, DNA metabarcoding, herbivore, population cycles, rodent

## Abstract

Herbivorous rodents in boreal, alpine and arctic ecosystems are renowned for their multi‐annual population cycles. Researchers have hypothesised that these cycles may result from herbivore–plant interactions in various ways. For instance, if the biomass of preferred food plants is reduced after a peak phase of a cycle, rodent diets can be expected to become dominated by less preferred food plants, leading the population to a crash. It could also be expected that the taxonomic diversity of rodent diets increases from the peak to the crash phase of a cycle. The present study is the first to use DNA metabarcoding to quantify the diets of two functionally important boreal rodent species (bank vole and tundra vole) to assess whether their diet changed systematically in the expected cyclic phase‐dependent manner. We found the taxonomic diet spectrum broad in both vole species but with little interspecific overlap. There was no evidence of systematic shifts in diet diversity metrics between the phases of the population cycle in either species. While both species' diet composition changed moderately between cycle phases and seasons, these changes were small compared to other sources of diet variation—especially differences between individuals. Thus, the variation in diet that could be attributed to cyclic phases is marginal relative to the overall diet flexibility. Based on general consumer‐resource theory, we suggest that the broad diets with little interspecific overlap render it unlikely that herbivore–plant interactions generate their synchronous population cycles. We propose that determining dietary niche width should be the first step in scientific inquiries about the role of herbivore–plant interactions in cyclic vole populations.

## Graphical abstract

This study investigates the diets of two key boreal rodents through DNA metabarcoding to evaluate whether their food intake varies systematically with their multi‐annual population cycles. The findings suggest that diet changes related to these cycles are marginal compared to overall diet flexibility and suggest that the broad diets, with little interspecific overlap, render it unlikely that herbivore–plant interactions generate their synchronous population cycles.

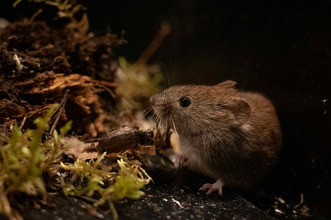

## 1 INTRODUCTION

Herbivorous rodents in boreal, alpine and arctic ecosystems are renowned for their multi‐annual population cycles (Elton, 1924; Hansson & Henttonen, 1985; Kendall et al., 1999). Decades of studies on rodent cycles have provided insights into many mechanisms involved in the dynamics and ecology of populations (Stenseth, 1999; Turchin, 2003). The generation of cyclic dynamics in rodents seems best explained by trophic interactions that act with a delay (e.g. reviewed in Andreassen et al., 2021; Berryman, 2002), including herbivore–plant interactions (Oksanen et al., 2020; Reynolds et al., 2016; Turchin & Batzli, 2001). Indeed, researchers have found that food plants play a critical role in the generation of cycles (Batzli & Pitelka, 1983; Prevedello et al., 2013), both based on experimental (Batzli, 1986; Gilbert & Krebs, 1981; Huitu et al., 2003; Johnsen et al., 2017) and observational studies on boreal and Arctic rodent populations (Boonstra & Krebs, 2012; Krebs et al., 2010; Laine & Henttonen, 1983). However, other studies have failed to find the necessary delayed effects of rodent‐plant interactions (Klemola et al., 2003), and the food‐plant hypothesis thus remains debated.

The food‐plant hypothesis concerning small rodent population cycles was recently systematically reviewed in Soininen and Neby (2024). The review found that it was best grouped into four sub‐hypotheses. One of these states that during high population densities, intensive herbivory by rodents reduces their preferred food biomass. Consequently, less preferred food plants are increasingly exploited (Lack, 1954). The less preferred food plants may provide fewer nutrients or pass on more defence compounds or toxins to the animal (Freeland, 1974; Jensen & Doncaster, 1999), thus limiting population growth. The recovery of the preferred food plants needs to be slow to keep rodent population growth slow and to produce a multi‐annual low phase. If we understand how diets change depending on the phase or density, we can better understand how plants affect small rodent and herbivore population dynamics (DeGabriel et al., 2014). Exploring changes in dietary diversity and composition across cycle phases is especially useful to assess the premises of this variant of food‐plant hypothesis. However, quantifying how rodents reduce food availability for themselves is challenging, as they may have diverse diets (Soininen et al., 2013, 2017). Also, since boreal rodents live cryptically under the snow for a large part of the year, our knowledge is incomplete regarding which species of food plants are critical for their health and reproduction during the winter season, when food availability may be limited—especially after population peaks (Huitu et al., 2008).

Generally, it is unclear how high animal densities and consequent intraspecific competition affect diet and diet diversity (Jones & Post, 2016). However, increasing population densities may lead to foraging on food items with lower quality/palatability (Stewart et al., 2011; Svanbäck & Bolnick, 2007). In the case of rodents, some studies have found evidence for a broader population‐level diet at high population densities (i.e. during the cyclic peak phase), both in terms of higher diet richness/diversity (Hansson, 1988) and increased use of less‐palatable food items (Bergeron, 1996; Gilbert et al., 2013; Hansson, 1986), while other studies did not find such patterns (Batzli & Pitelka, 1971; Bergeron & Jodoin, 1989; Hansson, 1969). One explanation for changes in population‐level diet diversity could be that rodent individuals utilise secondary habitats rather than widening their individual niches (Soininen et al., 2014). In any case, there is a risk of decreased food quality, which in turn may lower individual health (Forbes et al., 2015). Whether temporal shifts in the rodent diet could contribute to delayed density‐dependent or cyclic phase‐dependent population growth (sensu Stenseth, 1999) remains among the key questions in the study of small rodent population cycles (see review in Andreassen et al., 2021).

During the last decade, DNA metabarcoding for dietary analysis has become a popular approach (Taberlet et al., 2018), mainly due to its cost efficiency and superior taxonomic resolution compared to morphological methods (da Silva et al., 2019; Soininen et al., 2009; Valentini et al., 2009). Thus, it has become a common method for resolving the diverse diets of small rodents (Aylward et al., 2022; Lopes et al., 2020; Sato et al., 2019; Zhang & Han, 2021). DNA metabarcoding also has the potential to provide information about the relative biomass proportions of ingested food via different approaches (Deagle et al., 2019)—though neither is optimal in every study system (if in any). One approach is to divide each occurrence of a food item in each sample by the number of food items occurring in that sample (e.g. if plants A, B and C occur in a sample, each plant occurrence would be one‐third instead of one). The mean of these quotients per food item across samples gives the weighted per cent of occurrence (wPOO) as it weighs each occurrence according to the number of food items in each sample and thus reduces the influence of samples that contain a large number of food items (Deagle et al., 2019). Another approach would be calculating the relative frequencies of sequence reads in each sample (i.e. relative read abundance, RRA). Researchers commonly use the latter for its quantitative potential; however, RRA and ingested food biomass may not always correlate positively for several reasons (Lamb et al., 2019; Neby et al., 2021). We thus chose to include information on both metrics.

Here, we present the first study applying DNA metabarcoding to analyse the temporal variation in rodent diets across seasons and over the critical phases of a population cycle. Our study concerns the tundra vole *Microtus (Alexandromys) oeconomus* (Pallas, 1776) and the bank vole *Myodes (Clethrionomys) glareolus* (Schreber, 1780), which are among the most widespread, abundant and functionally important mammal species in boreal ecosystems in Europe (Boonstra et al., 2016). However, while bank voles are known as generalist browsers (Hansson, 1985), the tundra vole has been assumed to be a more specialist grazer (Batzli & Henttonen, 1990; Hansson, 1985), but see Soininen et al. (2013). Thus, the two species can be expected to have different dietary flexibilities. We test the general prediction that the transition between critical phases of the population cycle is associated with a change in diet in both species. More specifically, we test whether the transition from the Increase‐Peak phase to the Crash‐Low phase of the population cycle is associated with (1) an increase in population‐level diet diversity, (2) a dietary shift towards less preferred food plants and (3) that such phase‐dependent diets shifts should be most profound in the winter season when food is supposed to be most limiting.

## 2 MATERIALS AND METHODS

### 2.1 Study area

**FIGURE 1:**
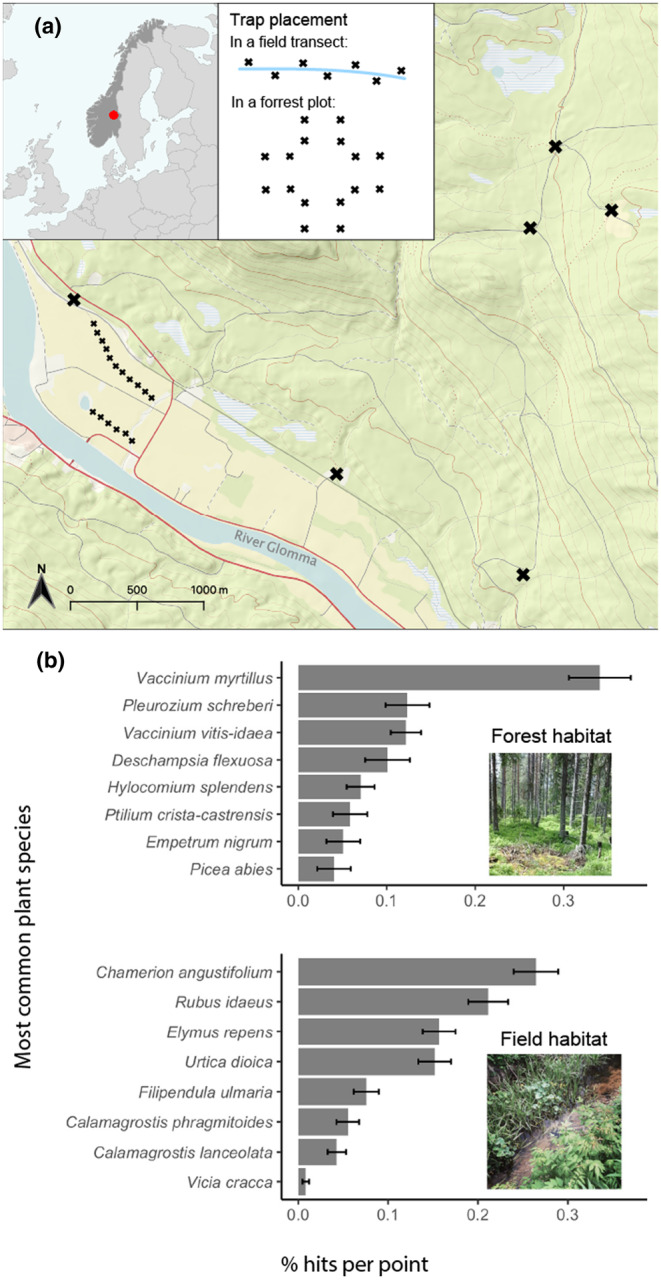
(a) Map of the study area at Evenstad, SE Norway with black crosses symbolising rodent traps (in inset) or trapping area. For trapping tundra voles (*Microtus oeconomus*) in the field transect, we positioned the traps on both side of agricultural ditches in transects. For trapping bank voles (*Myodes glareolus*) in the forest habitat, the traps were placed in a cross shape as in Ehrich et al. (2009). (b) The most common plant species in the forest and field habitat, as quantified by the Point Intercept method, described in Appendix S1.

The study area was near the Evenstad Research Station (61.4° N, 11.1° E, Inland Norway University of Applied Sciences, Figure 1), where both study species were known to exhibit cyclic population dynamics (Andreassen et al., 1998, 2020; Ims & Andreassen, 2000). The area is in the transition zone between the southern and middle boreal zones and is characterised by a sandstone‐dominated bedrock with additional sediment deposits along the river Glomma. The climate is relatively continental (Boonstra et al., 2016), with a mean annual air temperature of 3°C and precipitation of 571 mm (Evenstad weather station during 1974–2019, MET Norway, 2022). During this study, the snow melted between May 1st and 19th, and permanent snow cover commenced between October 25th and November 22nd (Appendix S1). During this period, the mean snow depth was approximately 65 cm, SD = 51 cm (Rena weather station, MET Norway, 2022).

### 2.2 Study design

Within the study area, we targeted the primary habitats of the two most common vole species: *forest* for the bank vole and along stream bank next to an agricultural *field* for the tundra vole. The study design was partly determined by existing vole population monitoring (Andreassen et al., 2020). Six plots were in the *forest* habitat (Figure 1), which was dominated by mature Norway spruce (*Picea abies*) and Scots pine (*Pinus sylvestris*) in the tree layer and with bilberry shrubs (*Vaccinium myrtillus*) and mosses (such as *Pleurozium schreberi*) in the understorey. In the *field* habitat, we selected two transects along drainage ditches, where grasses (e.g. *Elymus repens* L.) dominated the vegetation along the ditch, but also herbs (e.g. *Urtica dioica* L. and *Chamaenerion angustifolium*) and shrubs were common (e.g. *Rubus idaeus* L.) (Figure 1, Appendix S1).

**FIGURE 2:**
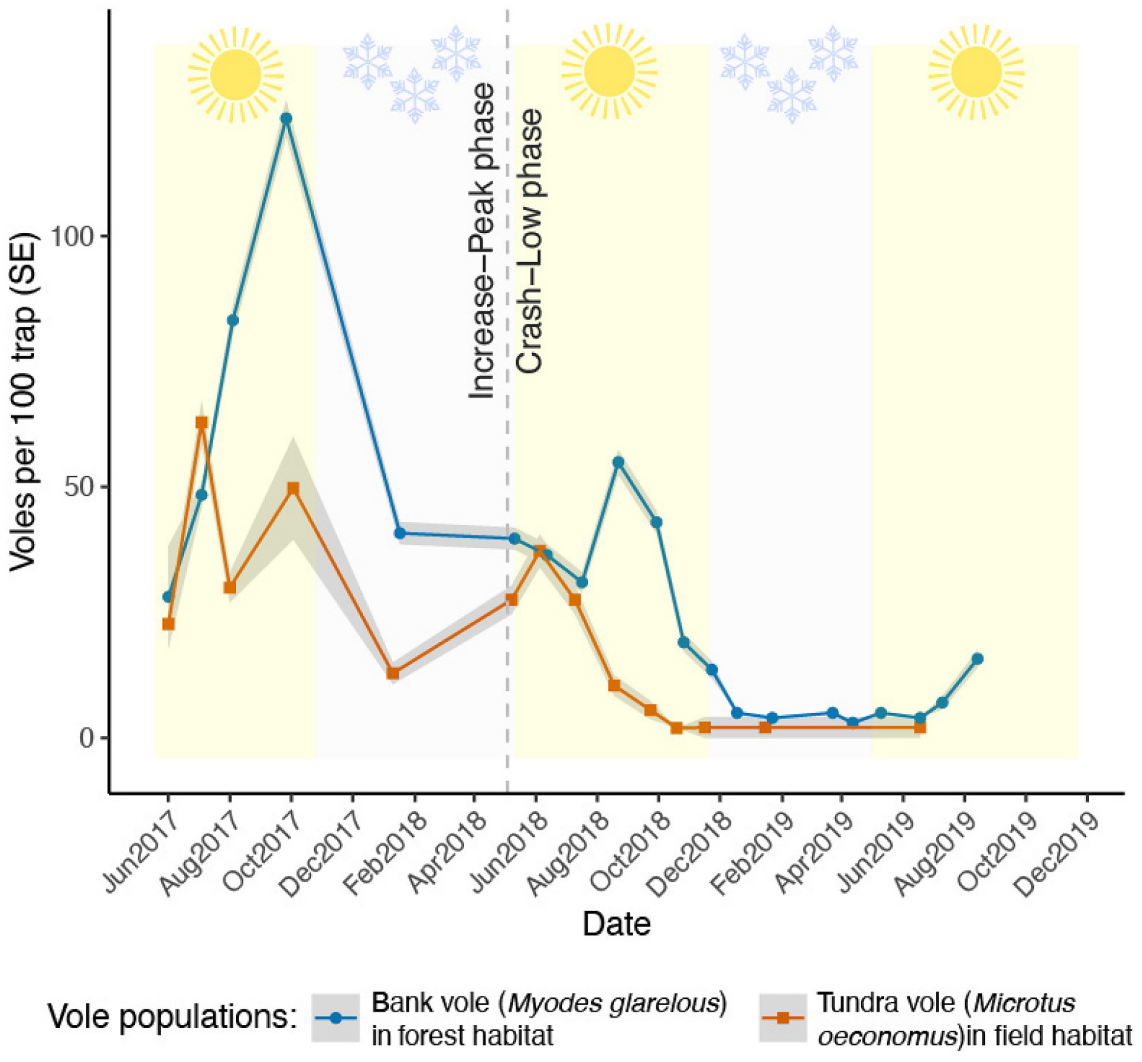
Population dynamics of bank voles and tundra voles in different seasons and phases of the cycle. The population size was estimated with the Robust Design with Closed Population Estimation model using the program MARK via RMark (Laake, 2013; White & Burnham, 1999) from capture‐mark‐recapture data (*n* = 6 plots and 96 traps for bank vole; *n* = 2 transects and 108 traps for tundra vole). Data are presented as number of voles per 100 traps, averaged per grid/transect ±SE (grey ribbons).

We collected data on vole population dynamics and diets in the forest plots and the field transects; in both summer and winter; over the years 2017–2019 with monthly to tri‐monthly intervals (Figure 2). Within each plot in the forest habitat, we placed 16 traps in a cross‐shaped design of 60 × 60 m (Figure 1, Ehrich et al., 2009). In the field habitat, we deployed the two transects (>350 m apart with intensive farming in‐between) along ditches consisting of 60 and 48 traps, corresponding to 1000 and 750 m, respectively (Norrdahl et al., 1993). The stations were positioned 15 meters apart on every other side of the ditch, 2 m from the ditch.

### 2.3 Population dynamics data

We monitored the vole population dynamics employing capture–recapture with Ugglan live traps (Grahnab, Sweden). We baited the traps with freshly cut carrots (2017–2019), apples (2019), oat seeds (2017–2019), peanuts and peeled sunflower seeds (2019). We activated the traps 12 h before the trapping session and checked them five times per session during the morning and evening (i.e. five secondary occasions per primary occasion sensu Kendall et al. (1995)). We left the traps deactivated and open until the next month's trapping session. For the diet analyses (see below), all faeces in the traps were removed prior to each trapping session to secure temporal precision, and the remaining bait and faeces were removed at the end of a trapping session to minimise the effect of artificial feeding. In order to trap voles during winter, we covered the traps with plastic boxes (38 cm × 59 cm × 39 cm) prior to the first snowfall, and we removed the boxes when the bare ground first appeared. We defined summer as time with snow‐free ground and winter as the time with permanent snow cover (i.e. winters were from 25‐Oct‐2017 to 11‐May‐2018 and from 20‐Nov‐2018 to 01‐May‐2019).

Each new individual weighing above 10 g was marked by injecting a small passive integrated transponder tag (7 mm length, Trovan, Ltd., UK) into the subcutis, allowing for radio frequency identification (RFID) during recaptures. We released all animals at their location of capture. We conducted the animal trapping in accordance with Norwegian laws and regulations concerning experiments with live animals, which are overseen by the Norwegian Food Safety Authority (FOTS 13908, 19475).

### 2.4 Diet data

We collected faecal samples in August and September 2017 and every month from January 2018 until August 2019 to analyse the diet of the voles. For each faecal sample, up to 10 faecal pellets were collected with a tweezer, placed in filter paper bags, and stored in plastic zip‐lock bags, pre‐filled with silica gel (Carl Roth, Germany). Whenever possible, we collected faeces directly from the traps during the first visit per trapping session, obtaining samples corresponding to the trapped individuals. We also collected faeces inside the traps when activating the traps before a month's trapping session, obtaining samples that we could not allocate to specific individuals—this was necessary in order to acquire enough faecal samples during low vole densities. We assessed the correspondence between the two sampling approaches and deemed them sufficiently similar to be analysed in a combined dataset (see Appendix S2). When we sampled faeces in traps without any captured animal, we assessed the species identity first visually based on the faeces' morphology. We then verified this initial assessment with a Sanger sequencing‐based DNA barcoding approach using the set of arvicoline‐specific primers Pro+/MicoMico (Alasaad et al., 2011), specifically designed to identify arvicoline mammals based on field‐collected faecal samples (Verkuil et al., 2018), see further details in Appendix S3.

For each vole species, we aimed to collect ten separate faecal samples with each sampling approach every month. However, since the number of available samples was very low during the cycle's low phase (Table 3 in Appendix S3), we adjusted the sample size to six separate faecal samples per month per species, to reduce the unbalance between high‐ and low‐density months. When more than six faecal samples were available for diet analysis for a given date/species, we prioritised samples that (1) contained normal/fresh‐looking faeces (i.e. not smeared into the filter bags), (2) were sampled at trapping stations furthest apart from each other and (3) had faeces morphologically matching either of the two target vole species. All selected faeces were analysed using DNA metabarcoding (Taberlet et al., 2018) to cover the voles' full diet spectrum.

DNA extractions from faecal pellets were carried out by Sinsoma GmbH (Innsbruck, Austria) using the Biosprint 96 DNA Blood Kit and a Biosprint 96 Robotic Platform (Qiagen, Germany). The protocol was carried out according to the manufacturer's instructions, except that (1) the lysis step consisted in adding 250 μL lysis buffer (TES buffer: Proteinase K (20 mg/mL) 19:1) in each sample before vortexing and overnight lysis at 58°C; and (2) DNA was eluted in 200 μL 1× TE buffer. DNA extraction negative controls (water instead of DNA) were systematically included.

We selected six complementary metabarcoding primer sets (see details and references in Table 1) to cover the diet spectrum of the two vole species, including plants, bryophytes, mushrooms/lichen, arthropods and other small invertebrates (Hansson, 1979; Hansson & Larsson, 1978; Smal & Fairley, 1980; Soininen et al., 2013). PCR reactions were carried out in a total volume of 15 μL using the AmpliTaq Gold 360 PCR Master Mix (Thermo Fisher Scientific, USA), 0.4 μL/15 mL of bovine serum albumin (BSA; Sigma‐Aldrich, USA), 0.5 μM of each primer and 2 μL of undiluted DNA. We initiated the PCR reaction by a denaturation step at 95°C for 10 min, followed by 35 cycles of denaturation at 95°C for 30 s, annealing for 30 s (see Table 1 for primer‐specific annealing temperatures), elongation at 72°C for 1 min, and a final elongation at 72°C for 7 min before a final hold at 15°C. Primers were synthesised with 8 or 9 bp sequence tags (https://github.com/pheintzman/metabarcoding) at each extremity in order to allow the assignation of sequences to each sample after sequencing. PCRs were run in duplicate with the primer sets targeting bryophytes, fungi and arthropods, or in triplicate with the primers targeting seed plants and eukaryotes. One PCR negative control (ultra‐pure Milli‐Q water instead of DNA) and one PCR positive control were included for each batch of 94 samples. For seed plants, PCR positive controls consisted of a mixture of six synthetic standard sequences with varying GC content, homopolymers, sequence length and concentrations (Table ESM3.1 in Appendix S3). PCR positive controls for bryophytes, fungi and eukaryotes consisted of a single synthetic DNA stretch used at a concentration of 1 ng/μL (Table ESM3.1 in Appendix S3), whereas PCR positive controls for the arthropod‐specific primers consisted of sequences from six known species of Coleoptera and Diptera, whose taxonomic identifications and DNA extracts were provided by the DNA Bank of the Natural History Museum in Oslo, Norway. A subset of PCR products was selected for the visual inspection of the amplified DNA using 1.5% gel electrophoresis. All PCR products were first pooled per primer set and purified using the QIAquick PCR Purification Kit (Qiagen, Germany). DNA concentrations from purified amplicon pools was then quantified using a Qubit 2.0 fluorometer and the dsDNA HS Assay kit (Invitrogen, Life Technologies, USA). Libraries were prepared from the purified pools (*n* = 11) using the KAPA HyperPlus kit (Kapa Biosystems, USA), and sequenced (2 × 150 bp paired‐end reads) on a HiSeq 4000 machine (Illumina, USA) at the Norwegian Sequencing Centre. The sequencing was carried out in two separate runs, and we merged the sequence reads data from both runs during the bioinformatic filtering process. Statistics on the processing steps are described in Appendix S3.

**TABLE 1:** PCR amplification primer sets used in the main diet study.

Common name taxonomic group	*Sper01* Spermatophyta	*Bryo01* Bryophyta	*Euka02* Eukaryota	*Fung01* fungi	*ZBJ‐ArtF1c/ R2c* Arthropoda	Pro+/MicoMico Arvicolinae
Gene region	trnL_P6‐loop (UAA)	trnL_P6‐loop (UAA)	18S V7 region	ITS1	COI Folmer region	mtDNA control region
Size range (bp)	10–220	41–87	36–892	68–919	50–200	Average 300
Annealing temperature (°C)	52	54	45	56	57	55
Reference	Taberlet et al. (2007)	Epp et al. (2012)	Guardiola et al. (2015)	Epp et al. (2012)	Zeale et al. (2011)	Alasaad et al. (2011), Haring et al. (2000)

Bioinformatic analyses were carried out using the Norwegian high‐performance computing cluster Saga (https://www.sigma2.no) and the ObiTools program (Boyer et al., 2016, http://metabarcoding.org/obitools). The forward and the reverse pair‐end reads were aligned and merged into a consensual sequence using *illuminapairedend* by considering the quality of the sequence data during the alignment and the consensus computation. Only alignments with scores >40 were kept for further analyses. For each primer set, sequences were assigned to samples with the *ngsfilter* command. Only sequences with a perfect match on tags and a maximum of two errors on primers were retained for further analyses. Primers and tags were cut off at this step. Then, we performed denoising by removing between‐sample chimeras with *obigrep*. The sequencing was carried out in two separate runs, but the sequencing reads data from both runs were merged at this step. Strictly identical sequences were clustered together using the *obiuniq* command, while keeping the information about their distribution among samples. Sequences shorter than 10 bp and/or occurring at ≤10 reads in the whole dataset were filtered out. Taxonomic assignments were carried out using *ecotag* and local reference databases, constructed for each primer pair by extracting the corresponding DNA region for the relevant taxonomic groups from the European Nucleotide Archive nucleotide library using the *ecoPCR* program (Bellemain et al., 2010; Ficetola et al., 2010). To improve the resolution of taxonomic assignations for plants, we also used local reference database for the Arctic‐boreal region (the ArctBorBryo database), containing 2280 reference sequences of the *trn*L P6 loop from 2001 different arctic and boreal vascular plants and bryophytes (Soininen et al., 2015; Sønstebø et al., 2010; Willerslev et al., 2014). Only sequences with unambiguous taxonomic annotation at the order level were included in the custom‐build reference databases. A unique taxon was assigned to each sequence. If several matches between the query sequence and the reference database were possible, the sequence was assigned to the taxon corresponding to the last common ancestor node of all the taxa in the NCBI taxonomic tree that best matched against the query sequence. These taxon were considered molecular operational taxonomic units (MOTUs) in the remaining analysis. Further details on the processing steps and changes in the number of samples, sequence reads, and MOTUs are described in Table ESM3.2 in Appendix S3. We carried additional data filtering using the ROBITools package (https://git.metabarcoding.org/obitools/ROBITools). PCR replicate outliers were discarded, with the assumption that they are the result of non‐functional PCR reactions. For this, we calculated the Euclidean distances of PCR replicates with their average (hereafter *dw*) and compared it against the distribution of pairwise dissimilarities between all average samples (hereafter *db*). Based on the expectation that PCR replicates from the same sample should be more similar than any two average samples (*dw* < *db*), we discarded PCR replicates lying outside the dissimilarity threshold; defined as the intersection of *dw* and *db* distributions. This process was repeated iteratively until no more PCR replicates were removed from the dataset. At the end of this procedure and in order to give equal weight to each replicate, the remaining PCR replicates were averaged for each sample. MOTUs representing less than 1% in at least one sample were discarded, thus effectively filtering out any tag jumps (tracked through the distribution and relative abundance of sequences from the PCR positive controls). PCR amplification success was confirmed by successfully retrieving all PCR positive control sequences. After further inspection of each of the datasets, we retained only MOTUs falling within the taxonomic range covered with each primer set (i.e. Spermatophyta for *Sper01*; Bryophyta for *Bryo01*, Agaricomycetes (mushrooms) and Lecanoromycetes (lichen) with *Fung01*, etc.). Based on their taxonomic identity, seed plants MOTUs were also classified into three broad functional groups—graminoids, forbs and shrubs. Eukaryote MOTUs were classified into five dietary categories: arthropods, lichens, mushrooms, bryophytes and plants. Each primer dataset was first analysed separately as described above. Second, taking the eukaryote primers dataset as a reference, we substituted the relative proportions of all dietary MOTUs detected with the eukaryote primers with the relative read abundance of dietary MOTUs amplified with the five other primers and identified with much greater taxonomic precision. The final dataset thus comprised the relative read abundances of plant, bryophyte, arthropod, lichen and mushroom MOTUs, allowing us to quantitatively compare differences in voles diet composition and diversity across seasons and population cycle phases. Finally, we normalised the sequence read abundances by dividing the number of reads for each MOTU by the total number of reads within each sample (i.e. relative read abundance, RRA). We also calculated the weighted per cent of occurrence (wPOO) (Section 1; Deagle et al., 2019; Tollit et al., 2017) for MOTUs whose RRA within a sample was above 1%. We calculated wPOO by dividing each MOTU in a sample by the number of MOTUs occurring in that sample and averaging these quotients per MOTU across samples.

### 2.5 Statistical analyses

#### 2.5.1 Population dynamics

To estimate monthly abundances per grid/transect for each species, we used a closed population robust design model (Pollock, 1982) with package RMark and program MARK (Laake, 2013; White & Burnham, 1999). Due to low sample sizes during the low phase, we aimed for simplicity and kept capture/recapture probability equal between species and constant through time within a primary occasion (i.e. trapping session). We selected the best models based on Akaike's Information Criterion, corrected for small sample size (AICc; Burnham & Anderson, 1992, see Appendix S4). We used alpha level of .05 unless stated otherwise.

#### 2.5.2 Diets

To investigate a potential increase in population‐level diet diversity, we analysed diet diversity using Hill numbers (^q^D, *q* = 1) (Hill, 1973). We modelled taxon diversity as a function of the cycle phase (Increase‐Peak, Crash‐Low, for definitions, see Results) and/or the season (summer, winter) by fitting and testing analysis of variance (ANOVA) using permutation tests with the package lmPerm (Wheeler et al., 2016).

To test for a possible dietary shift towards less preferred food items, we analysed phase and season dependency on voles' diet composition (RRA). The RRA matrices were Hellinger transformed (i.e. square roots of the relative abundances) to downweigh the highly abundant genera while avoiding overweighting rare ones (Legendre & Gallagher, 2001). To derive a more holistic and robust understanding of the dataset, we used a permutational multivariate analysis of variance (PERMANOVA) and a non‐scaled redundancy analysis (RDA) to evaluate differences in diet composition (package ade4 and vegan, Dray & Dufour, 2007; Oksanen et al., 2012). RDA is a constrained linear ordination method that explains the relationship between explanatory variables (as opposed to unconstrained methods such as principal component analysis, PCA). Similar RDA/PCA plots indicate that RDA explains the variation well. For bank voles, we included the interaction between phase and season as a four‐level factor and sampling grid as a covariate (Dray & Dufour, 2007; Oksanen et al., 2012). Due to low sample size in the interaction Crash‐Low phase: Winter (*n* = 4) on the tundra vole data we only included additive explanatory variables (phase, season). We used the packages Factoextra, ggvegan, ggplot2 and veganUtils to aid visualisation (Kassambara & Mundt, 2017; Simpson, 2015; Wickham, 2011).

Since both vole species are primarily herbivorous (Hansson, 1985; Tast, 1974; our Eukaryote class level diet data), we further investigated the plant functional groups within the diet and how their relative proportions vary with the cycle phase and the season. Broader groups such as graminoids, forbs and shrubs are relevant for comparisons with previous, lower‐resolution dietary studies (e.g. Batzli & Lesieutre, 1991; Hansson & Larsson, 1978) and also enable to take into account the contribution of low‐occurrence taxa. We only used seed plants dataset (*Sper01* primers) and ran analyses separately for RRA and wPOO. For each vole species, we fitted regression models (Brooks et al., 2017) to explore how the abundance of plant MOTUs was affected by phase, season and plant functional group identity (graminoid, forb, shrub), all used as additive predictors. For bank voles, we also included the sampling grid identity as a random effect. We used package DHARMa for residual diagnostics (Hartig & Hartig, 2017) throughout. We estimated marginal contrasts to identify whether the proportions of the functional groups differed significantly between cyclic phases or seasons (Makowski et al., 2020).

## 3 RESULTS

### 3.1 Population dynamics

Our study period encompassed the Increase‐Peak phase and the Crash‐Low phase of the typical 4‐year vole population cycle in the study area (Figure 2; see also Andreassen et al., 2020 for more long‐term monitoring data from the same study area). Both species peaked during the 2017 summer season. The subsequent decline over the winter season 2017/2018 (the two species normally do not breed in winter) was relatively moderate, resulting in higher abundances at the onset of the summer in 2018 than in 2017. Hence, we defined summer 2017 and winter 2017/2018 as the (late) Increase‐Peak phase for both species (Figure 2). Due to the marginal increase in the bank vole numbers and the strong decline in the tundra vole population during the 2018 summer season, and the subsequent declining and eventually very low abundances of both species (Figure 2), we defined the period encompassing summer 2018, winter 2018/2019 and summer 2019 as the crash‐low phase (i.e. representing an H‐decline according to Chitty, 1955).

### 3.2 Diets

**FIGURE 3:**
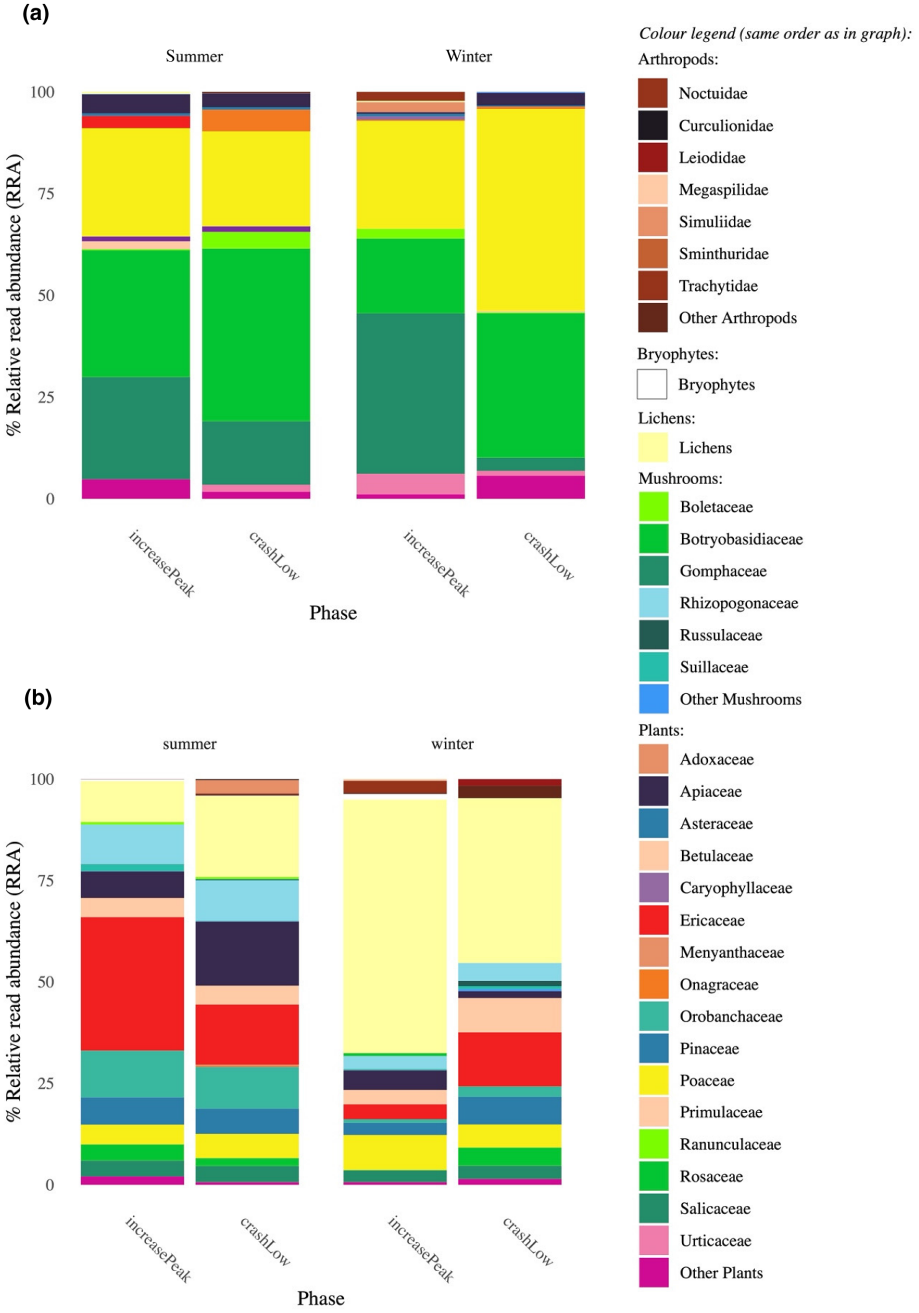
The full diet spectrum of the two vole species. The vertical axis estimates of the relative importance of taxonomic families over other potential food items in the diet of (a) tundra voles and (b) bank voles. Diet composition was estimated with DNA metabarcoding by combining the complementary primer sets in Table 1.

High‐throughput sequencing provided in total 170,272,750 reads. The best taxonomic rank at which dietary MOTUs were identified differed among primer sets: species level (*Fung01*), genus level (*Sper01* and *Bryo01*), family level (*ZBJ‐ArtF1c/R2c*) and class level (*Euka02*). The bank vole dataset comprised between 73 and 131 samples and between 35 and 638 MOTUs for the different primer sets (Appendix S3). The tundra vole dataset comprised between 16 and 69 samples and between 16 and 552 MOTUs for the different primer sets (Appendix S3). Both vole species had diverse diets dominated by plants, but with little interspecific overlap (Figure 3).

**FIGURE 4:**
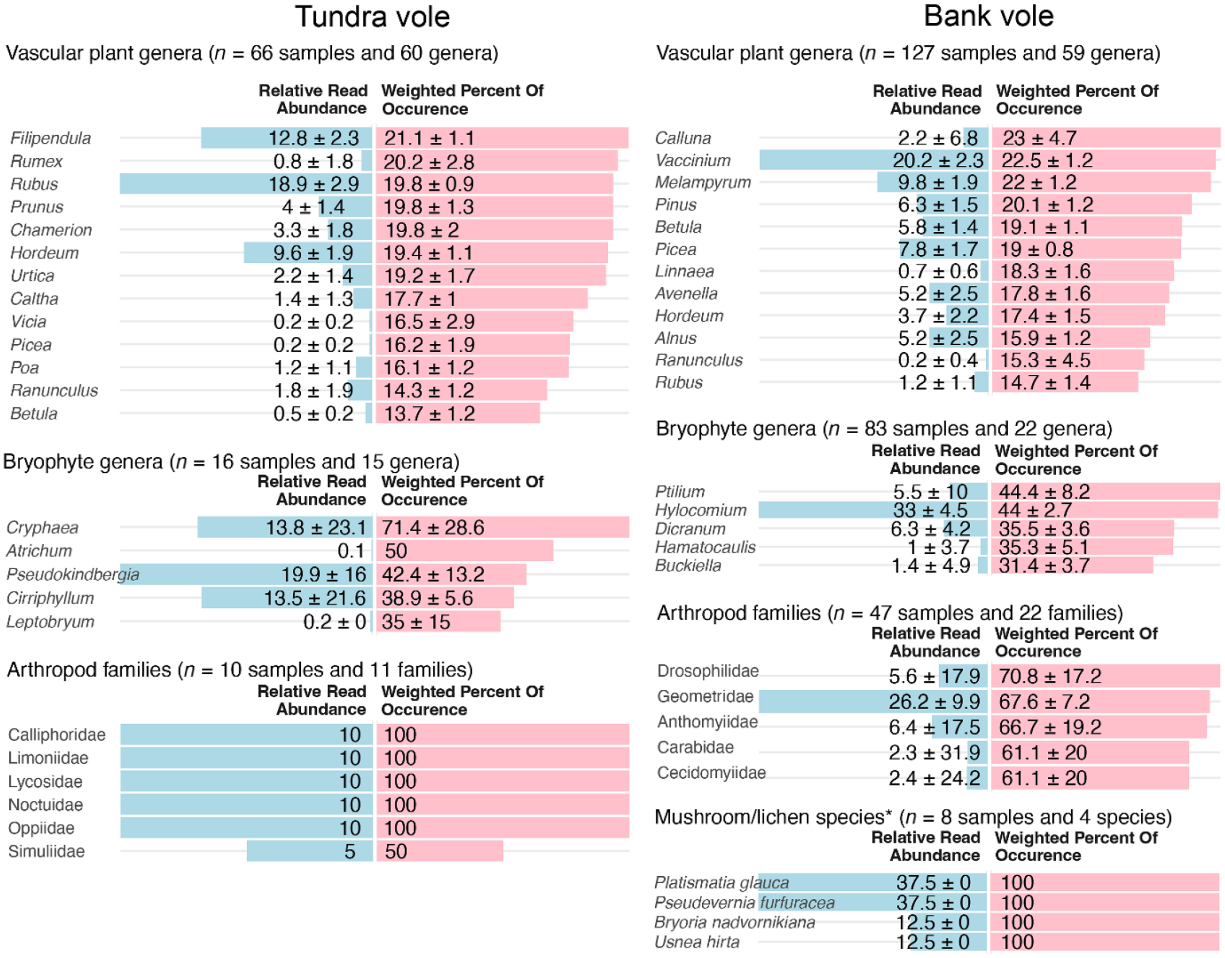
Diet composition of bank and tundra voles, based on DNA metabarcoding analysis. Primer sets included are: seed plants *Sper01* (*trn*L), bryophytes *Bryo01* (*trn*L), fungi *Fung01* (ITS1), arthropods *ZBJ‐ArtF1c/R2c* (COI) (Table 1). For each primer set, relative read abundance (RRA) and frequency of occurrence (wPOO) are included for the most common taxa (mean across individual samples ± standard error). Occurrences were included if a MOTU contributed >1% of the sequences in a sample and was detected in >5% of the samples. The number of samples for each primer set corresponds to the samples passing all quality controls after bioinformatic filtering. The taxonomic level presented differs between primer sets depending on the gene marker resolution and the quality of the sequence reference database. Samples with RRA without standard error estimate and 100% wPOO correspond to samples for which a single MOTU was detected (e.g. the arthropod family Calliphoridae). An overview of RRAs for all taxonomic levels is presented in Appendix S5. *Mushrooms (Agaricomycetes) and lichens (Lecanoromycetes) were amplified with the *Fung01* (ITS1) primer set.

In tundra voles, with the seed plants dataset (*Sper01* primers), we detected 16 plant genera occurring in >50% of the samples (Appendix S5). Based on RRA and wPOO together, the tundra vole plant diet was diverse in its functional groups with graminoids such as *Alopecurus* sp. and *Hordeum* sp., forbs such as *Filipendula* sp., and *Salix* and *Rubus* shrubs (Figure 4). Overall, bryophytes (*Bryo01* primers) were detected only in a small number of samples (*n* = 16), mainly the genus *Cirriphyllum* and had a low contribution to the tundra vole's diet (Figure 4). No mushrooms or lichen taxa (*Fung01* primers) were detected in the tundra vole diet. Arthropods (COI *ZBJ‐ArtF1c/R2c* primers) were retrieved from 10 samples, with the most common MOTUs matching dipterans, lepidopterans and arachnids (Figure 4), while no arthropods were detected with the broader eukaryotes primers (18S *Euka02*). On average 17 seed plants genera were detected per sample (SD = 5, range 7–31, *n* = 66), with diet comprising a total of 59 genera (Appendix S5). The Hill diversity index (^1^D) for seed plants was 4.2 ± 1.7SE (for MOTUs identified at the genus level) and 4.8 ± 20.3SE (for all MOTUs).

In bank voles, seed plants and lichen were the major dietary components (Figure 3). Seed plants comprised nine genera occurring in >50% of the samples, but many more occurred sporadically (Appendix S5). The diet was diverse including large portions of shrubs (*Vaccinium* sp., *Picea* sp., *Pinus* sp., *Betula* sp.), but also graminoids such as *Alopecurus* sp. and *Avenella* sp., and forbs such as *Anthriscus* sp. and *Melampyrum* sp. (Figure 4). Lichen (amplified with the *Fung01* primers) included mainly fruticose species such as *Platismatia glauca*, *Pseudevernia furfuracea*, *Bryoria nadvornikiana and Usnea hirta* (Figure 4). Bryophytes were detected in the bank vole diet, here amplified with both the *Bryo01* and the *Euka02* primer sets. *Hylocomium* were the most frequently detected genus, but various other genera from the Hypnales order were also detected (Figure 4). Dipterans such as Anthomyiidae as well as lepidopterans from the Geometridae family were the most common arthropods detected and were more abundant in the bank vole diet compared to the tundra vole (Figure 4). For bank voles, on average 15 plant genera were detected per sample (SD = 5, range = 4–34, *n* = 127), with a total of 58 genera (36 species, 35 families). The Hill diversity index (^1^D) for seed plants was 3.6 ± 1.8SE (for MOTUs identified at the genus level) and 4.2 ± 0.2SE (for all MOTUs).

### 3.3 Sources of variation in diets

**FIGURE 5:**
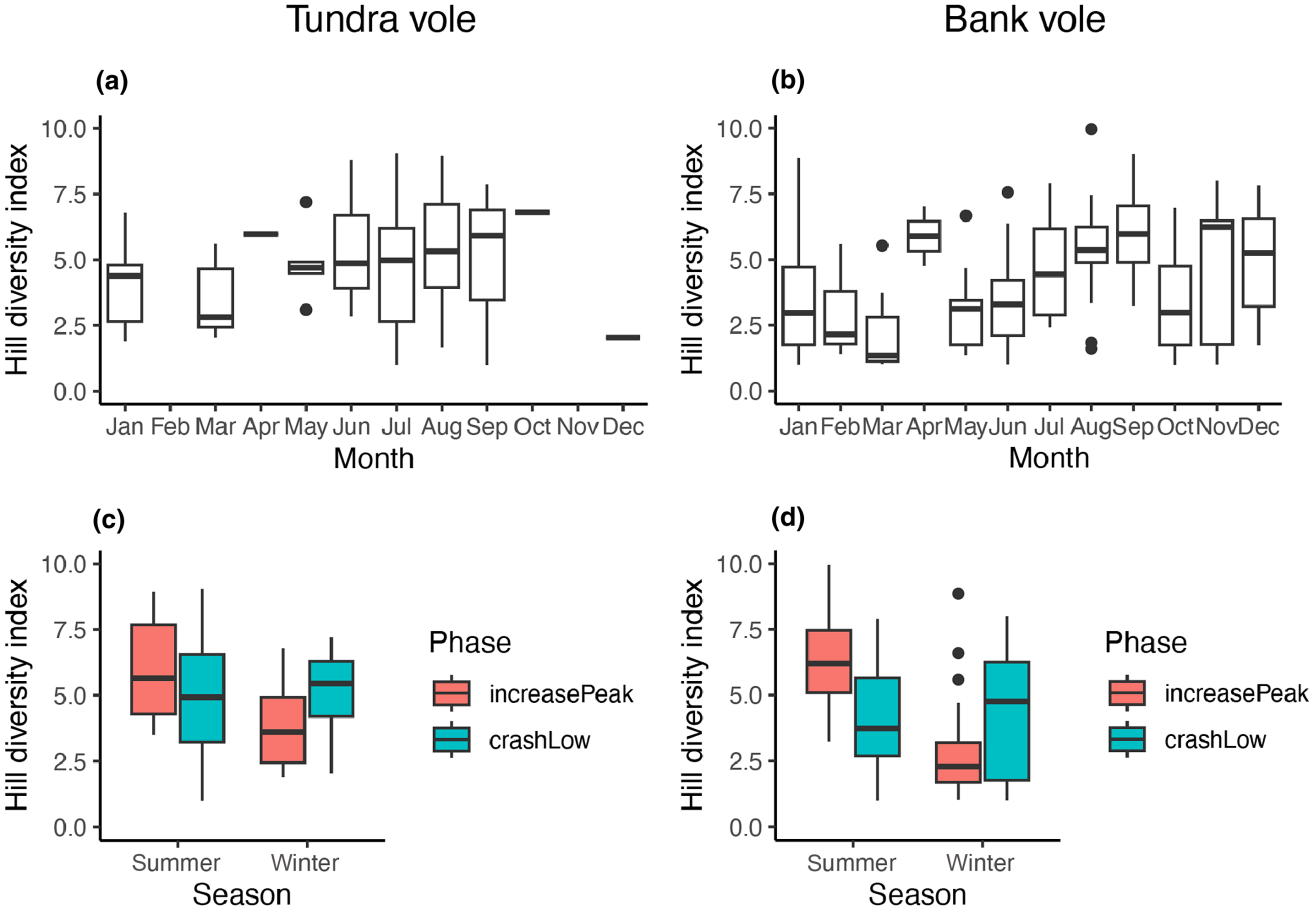
Boxplots with 95% confidence interval (CI) for Hill's diversity index using MOTUs detected in the diets of tundra voles (left) and bank voles (right) over time (panels a and b), and according to season and cyclic phase (panels c and d).

We did not detect an increase in population‐level diet diversity in the transition from the Increase‐Peak phase to the Crash‐Low phase. In tundra vole diets, this is supported by the permutation test using the diversity index as the response variable and season and phase as explanatory variables, resulting in a *p*‐value of .46. Contrastingly, in bank voles, the results of the permutation tests revealed a significant seasonal influence (*p* < .001) and season interacting with phases (*p* < .001). However, these factors accounted for only a small portion of the observed variation leaving 78% (residuals) unexplained by the model. Diet diversity index tended to be highest in winter months for bank voles, while we cannot make such claim for tundra voles due to limited sample sizes (Figure 5).

We could not detect a difference in diet composition between the cycle phases. But season and phase (PERMANOVA, *p* < .001 and *F* > 2.04) had a significant effect on the seed plants diet composition (RRA matrix), except for the effect of seasonality in tundra voles which was non‐significant, supplemental material Table 6.1 in Appendix S6. However, the redundancy analysis (RDA) shows little contribution from seasonality and phases in either species; the models on the diet of tundra voles and bank voles had adjusted *R* ^2^ of 7% and 20%, respectively.

**FIGURE 6:**
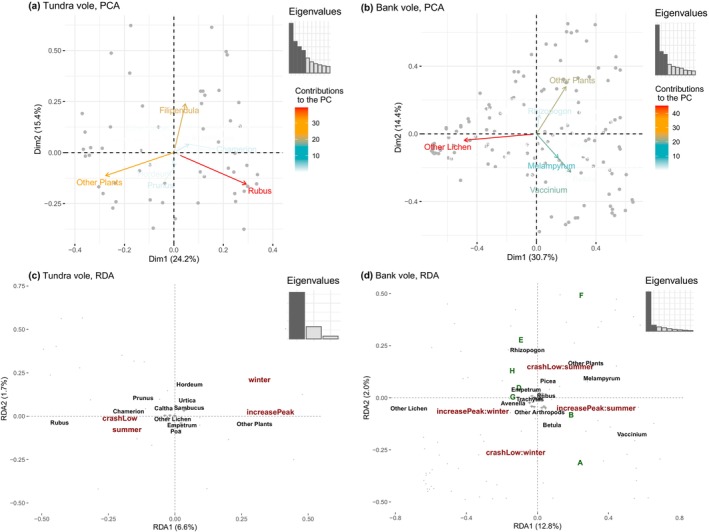
Multivariate analyses of vole diet composition, based on the relative read abundance (RRA) of dietary MOTUs from DNA metabarcoding. Upper panels (a, b) show PCA, and lower panels (c, d) show RDA with the effect of the cycle phase, the season and the trapping site. Bank voles are represented on the right (b, d) and tundra voles on the left (a, c). Grey points represent individual samples in all subplots. *X*‐axes represent the first PCA/RDA axis, *Y*‐axes represent the second PCA/RDA axis, and all axes are labelled by the per cent of explained variation. The eigenvalue plots show the contribution of the PCA/RDA axes in explaining variation in diet composition (Table 6.1 in Appendix S6). Explanatory variable names have been abbreviated: In tundra vole data, season (Summer (*n* = 50), Winter (*n* = 18)), phase (Increase‐Peak (*n* = 20), Crash‐Low (*n* = 48)) and their interaction in bank vole data (Increase‐Peak/Summer (*n* = 13), Increase‐Peak/Winter (*n* = 26), Crash‐Low/Summer (*n* = 61), Crash‐Low/Winter (*n* = 26)). The labels of any taxa contributing less than 0.1 on the first two axis are hidden or replaced by (+) symbols to avoid overlapping names. Note that the PCA and RDA plots have different rotation. If a taxon in the RDA plots is close to an explanatory variable vector, then they were positively correlated.

Due to the lack of clear seasonal and cycle phase patterns in diet diversity and composition, we could not test our third hypothesis about the phase‐dependent diet shifts in winter when food is supposed to be most limiting. Season does seem to be playing an important part in the RDA (tundra vole, adjusted *R* ^2^ = 7%), as variation in diet composition was driven by Axis 1 (6.6%), which was associated with the Increase‐Peak phase and the winter season (Figure 6c). The opposite direction of Axis 1 was rather associated with summer season and crash‐low phase as well as with the plant genera *Rubus*, *Chamerion* and *Prunus* (Figure 6c).

Season and cycle phases had a significant effect on the bank vole plant diet composition but also had a low explanatory power compared to overall variation. In the RDA (adjusted *R* ^2^ = 20%), the first two axes explained limited amount of the variation (12.8% and 2.0% for Axis 1 and 2, respectively, Figure 6d). However, the explanatory variables were separated into all the expected groups, that is Winter/Increase‐Peak, Winter/Crash‐Low, Summer/Increase‐Peak and Summer/Crash‐Low diets. Season was mainly explained by first axis and thus with the largest contributions, while phase was mainly explained by the second axis. Lichen and the graminoid genus *Avenella* were clearly associated with Increase‐Peak/Winter, while the shrub genus *Vaccinium* was associated with Increase‐Peak/Summer. The Salicaceae family was associated with Crash‐Low/Winter while the hemiparasite *Melampyrum* sp. with Crash‐Low/Summer. Overall, the biplots (and compositions) showed substantial individual variation in plant diet composition in both vole species, that is the grey dots representing individual's diets (Figure 6) show spatial differences (between local sampling sites) that were as large as the temporal differences across seasons and phases (Figure 6).

**FIGURE 7:**
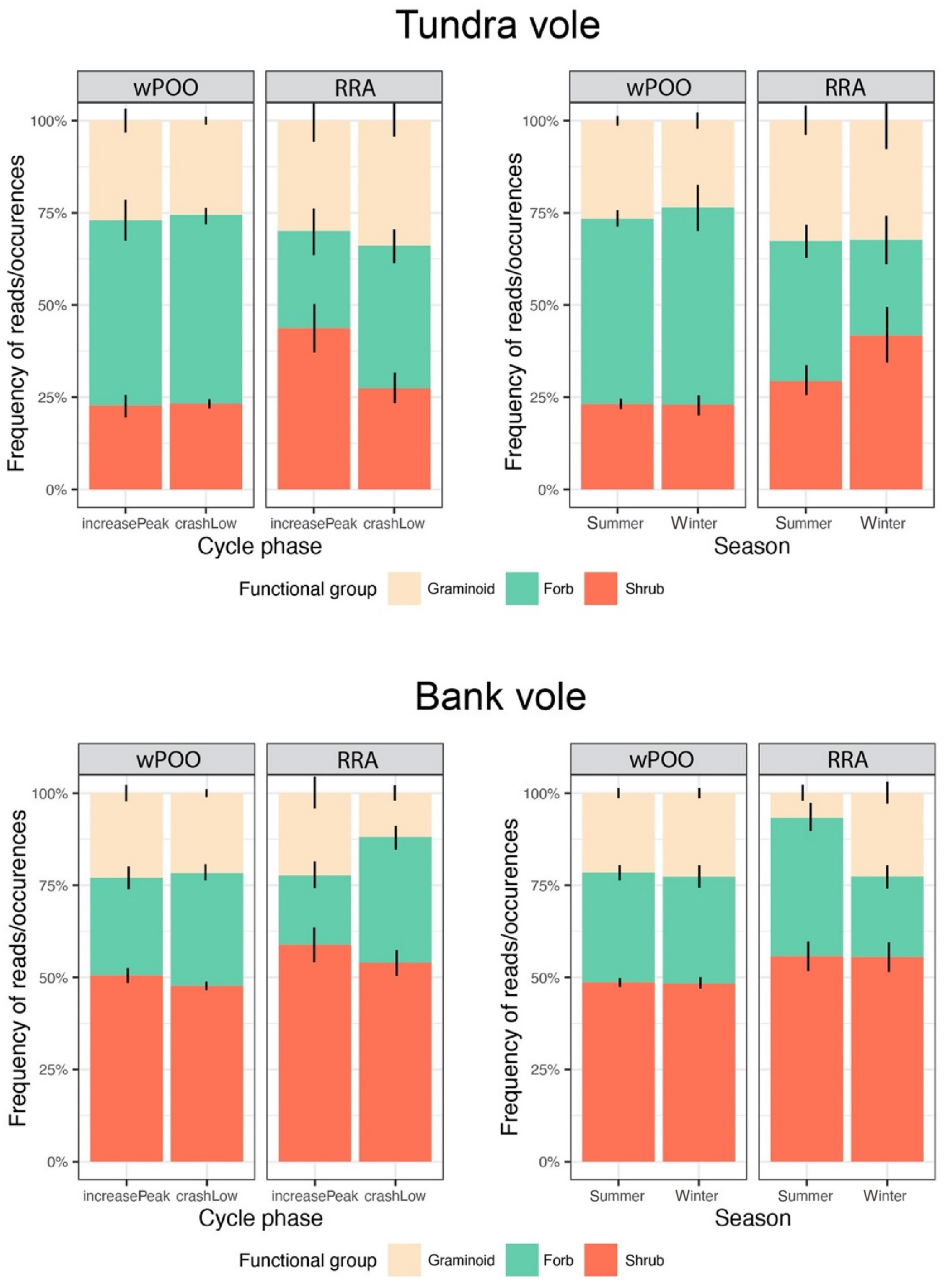
Comparison of plant functional groups across cycle phases (left‐hand panel) and seasons (right‐hand panel) for the two vole species, based on MOTUs, amplified with the *Sper01* primer set. Both relative read abundance (RRA) and weighted per cent of occurrence (wPOO) are presented. Black vertical lines correspond to the standard error.

The marginal contrasts of the regression models did not detect a significant effect of season and cycle on the proportions of the plant functional groups (graminoids, forbs and shrubs) (Table 6.2 in Appendix S6). However, the estimated functional group proportions (Figure 7) displayed weak patterns that were consistent across the two vole species: The use of forbs tended to be lower during winter compared to summer and lower during Increase‐Peak than Crash‐Low. In contrast, the use of shrubs tended to be higher during Increase‐Peak than Crash‐Low.

## 4 DISCUSSION

The present study is the first to use DNA metabarcoding to assess whether vole diet composition and diversity change systematically according to season and critical phases of a population cycle. While we observed tendencies for moderate shifts in diet composition in bank and tundra vole diets, both between population cycle phases and seasons, we found no evidence for similar shifts in diet diversity. Thus, changes do occur over time in vole diet composition, although temporal change at the population level appeared to be minor compared to dietary variation among individuals. Overall, this study indicates that the variation in diet that could be attributed to cyclic phases is marginal relative to the overall diet flexibility. Hence, it seems unlikely that temporal variation in diets was driving the transition between Increase‐Peak and Crash‐Low phases of the population cycle. It is also noteworthy that the two vole species displayed synchronous population dynamics, suggesting that a common plant factor did not cause this synchrony.

### 4.1 Vole species

Although the tundra vole has been described as a relatively specialised grazer of graminoids and forbs (Naughton, 2012), our study points to a wider diet in this species; for instance, it included substantial amounts of shrubs (among other food items). This finding is supported by previous research, which reports that the tundra voles browse *Salix* shrubs (Ravolainen et al., 2013; Soininen et al., 2013). The tundra vole diet consisted mainly of seed plants, with a variety of herbaceous monocotyledons and dicotyledons and with considerable variation in diet composition among individuals. The proportion of monocotyledons was smaller than dicotyledons, as also found by Soininen et al. (2013), in contrast to earlier studies employing other approaches for characterising the diet (Batzli & Henttonen, 1990; Batzli & Jung, 1980; Tast, 1974). Traditional methods such as microscopic examination of stomach contents are known to overestimate the proportion of taxa such as grasses compared to forbs (Anthony & Smith, 1974). Yet, this discrepancy may also be explained by habitat‐specific differences in diet, as suggested by Soininen et al. (2013).

The plant diet of bank voles was also diverse and with large individual variation covering all plant functional types considered. This fits well with the bank vole being a habitat generalist, that is inhabiting a wide range of habitats (Abt & Bock, 1998; Bonacchi et al., 2017; Dróżdż, 1966; Hjältén et al., 1992; Savola et al., 2013; Shaw et al., 2013; Watts, 1968). *Vaccinium* shrubs were the most abundant and frequently occurring plant genus, as also described by earlier studies (e.g. Hansson, 1979; Hansson & Larsson, 1978), while forbs and grasses collectively were as abundant as shrubs in the diet. We detected bryophytes in low proportions, similar to previous observations (Hansson, 1979; Hansson & Larsson, 1978; Hjältén et al., 1996). We found fruticose lichens associated with tree bark and arboreal tassels hanging in trees to be an important component of the bank voles' diet, while mushrooms (Agaricomycetes) were rare. However, mushrooms could been underestimated in our case, as the bulk of fungi‐matching sequences were assigned to the more abundant coprophilous fungi, commonly found in animal faeces (Richardson, 2001). Viro and Sulkava (1985) found hanging lichens (e.g. *Bryoria* sp.) in 28%–54% of the bank vole diet in all seasons; the lower range correspond well with our observations during summer, and the higher end of the range to the winter. Similar proportions are described in Hansson and Larsson (1978), and also other studies confirm lichens as part of bank vole feeding (Hansson, 1985; Nybakken et al., 2010). Previous reports describe other fungi or mushrooms mainly eaten in summer‐autumn, between just 8%–10% corroborating with our results (Heroldová, 1994; Viro & Sulkava, 1985); to high proportions (Hansson, 1979; Hansson & Larsson, 1978). Few studies include taxonomic details, however, in Kataržytė and Kutorga (2011) several genera and families are detected, including Russulaceae and Boletaceae also found in our study. The latter, as well as Rhizopogonaceae (the most common fungi family in our dataset) belong to the Boletales order, described as being dominant in bank vole diets (Kataržytė & Kutorga, 2011). In addition, we detected different families of arthropods (mostly flies and moths) occurring in both vole species' diets, but mainly in the bank vole data. As most previous descriptions of vole diets were made with very coarse taxonomic resolution due to methodological limitations (Canova & Fasola, 1993; Hansson, 1979; Heroldová, 1994; Smal & Fairley, 1980; Viro & Sulkava, 1985), it is difficult to assess whether arthropod consumption is common and deliberate. In any case, such high‐protein food might be a functionally important component of vole diets, even in low abundance. Overall, it is evident that bank voles have diverse diets—even in a relatively homogenous habitat dominated by bilberry and spruce.

As we sampled the vole species in different habitats, dietary diversity is not necessarily comparable (MacArthur & Pianka, 1966). However, the number of plant species (i.e. diet richness) was high for both vole species. The plant richness and range of plant functional groups in the tundra vole diet observed in a boreal field habitat are similar to what was found in tundra voles from an Arctic meadow habitat using a similar methodology (Soininen et al., 2013). Yet, while the number of food items in both vole species' diets was high in our study, most items were detected only in limited proportions. When only comparing seed plants diversity, we found that the tundra vole had slightly higher overall plant diet diversity than the bank vole, though the two species had similar plant diet richness. However, when considering the use of non‐plant food, the bank vole appeared to have the greatest diet spectrum of the two species.

### 4.2 Seasons

We found moderate variation in both vole species' diets between seasons. For tundra voles, the clearest seasonal patterns were a wintertime reduction of forbs and an increased use of *Salix* shrubs, in line with previous findings (Tast, 1966) and matching with seasonal availability. For bank voles, the seasonal changes were most apparent in terms of increased proportions of graminoids over forbs during winter, as also found by Viro and Sulkava (1985); and an increase in the use of lichens during winter, also identified in previous studies (Ecke et al., 2018; Hansson, 1985; Hansson & Larsson, 1978; Viro & Sulkava, 1985). Increased arboreal feeding may be beneficial if it helps voles to avoid subnivean weasel predation (Mäkeläinen et al., 2013), but it can also be detrimental in terms of increased exposure to arboreal predators, low temperatures and less‐nutritious food (Mäkeläinen et al., 2013) with potentially harmful secondary compounds (Ecke et al., 2018; Nybakken et al., 2010).

### 4.3 Cycle phases

We detected no clear diet shifts (in composition or diversity) associated with the transition from the Increase‐Peak to the Crash‐Low phases of the population cycle. We also find that the modest changes observed in diet composition were not driven by plants with known low palatability. We did observe large inter‐individual variation, also confirmed by previous diet studies (Soininen et al., 2013; Viro & Sulkava, 1985). The dietary flexibility of the two study species is further underlined by the fact that the spatial differences between local sampling sites were as large as the temporal differences across cyclic phases and seasons (i.e. sample sites distributed widely in Figure 5d). Hence, the dietary flexibility indicates that the consistent phase‐dependent tendencies are moderate at best. This is also supported by a recent study that found that plant–vole abundance relations were inconsistent over two consecutive population cycles (Soininen et al., 2018).

### 4.4 Flexible diets and population dynamics

A key finding of the present study is that the two vole species seem prone to feed upon a wide range of taxa and, thus, were very flexible and diverse in their diets. Hence, categorisations of the tundra vole as a specialised grazer and the bank vole as a more generalist browser are unwarranted. Thanks to the application of highly sensitive methods for diet analysis such as DNA metabarcoding, both species can be considered generalist herbivores within their respective habitats.

Several studies of plant–rodent interactions have suggested that fluctuations in the quality of single plant species could drive population cycles, for example *Vaccinium myrtillus* for *Myodes* spp. (Dahlgren et al., 2007; Selås, 2020), *Carex bigelowii* for *Lemmus lemmus* (Seldal et al., 1994) or *Deschampsia caespitosa* for *Microtus* spp. (Massey et al., 2008). One reason for this is that food quality analyses of single plant species are obviously more easily performed than analyses of foodscape quality (Petit Bon et al., 2021; Vonthron et al., 2020). However, the present study and earlier DNA metabarcoding studies (e.g. Soininen et al., 2009, 2013), show that boreal and arctic vole species with cyclic dynamics do not seem to use a single resource but have diverse and flexible diets. In such cases, we argue that interactions with a single plant species are not likely to underlie their population cycles.

The winter season is regarded as the bottleneck of food availability for herbivorous animals in northern food webs (Fauteux et al., 2021), but we lack knowledge on the below‐snow food availability of small mammals. We did not assess plant forage quality here, nor can dietary metabarcoding provide information about the plant tissue (e.g. root, bark, fruit, seed or leaf) or life stage (e.g. adult, larvae or pupa) ingested, there still could be hidden patterns in the feeding of the two vole species. This challenges some of our interpretations of the seasonal variation we observed. It is likely that several of the identified plants in this study are ingested not only as green leaves, but also as roots, bark, seeds, winter buds and berries (Batzli & Henttonen, 1990; Canova & Fasola, 1993; Hansson, 1979; Heroldová, 1994; Viro & Sulkava, 1985), thus contributing to the increased proportion of shrubs in the tundra vole winter diets. Shrubs such as *Vaccinium* produce nutritious berries, while coniferous such as *Picea* sp. and *Pinus* sp. produce vast amounts of seeds and seedlings, which are much more easily digested than the woody parts of adult trees. Furthermore, we cannot exclude the possibility that shifts in forage quality occurred in synchrony with vole population dynamics due to, for example drought stress or past herbivore pressure (Laine & Henttonen, 1983; Selås et al., 2018). The DNA metabarcoding in the present study suggests only modest differences among summer and winter diets. This may suggest that multiple diets exist within a similar nutritional niche (Hecker et al., 2021). Other methods in combination are thus needed to assess whether food quality or quantity in winter causes population crashes in voles. However, it should be noted that population crashes that take place in the midst of the plant growth season (summer declines; Hansson & Henttonen, 1985), as in the present study (Figure 2), are not likely to be caused by limited food quantity.

Indeed, according to general theory on consumer–resource interactions (e.g. Murdoch et al., 2013; Turchin, 2003), profound cyclic oscillations are expected only when the consumer is specialised on a specific resource (i.e. stenotopic consumers). Consumers with flexible diets will not be expected to have the kind of tight coupling with the dynamics of a single resource that acts to destabilise their population dynamics. While this conjecture has guided empirical studies of predator–prey and host–parasitoid interactions (Berryman, 2002), it appears to have had limited influence on the study of herbivore–plant interactions in rodents. Indeed, determining whether an herbivore is a generalist with a diverse and flexible diet, or a specialist with a narrow and inflexible diet, ought to be the first step towards an understanding of the role of herbivore–plant interactions in cyclic vole populations. Metabarcoding of faecal DNA for a wider array of rodent populations—ranging from those exhibiting low‐amplitude, seasonal dynamics (e.g. temperate *Myodes* sp. populations) to those with high‐amplitude, multi‐annual cycles (e.g. Arctic *Lemmus* sp. populations)—could be a good approach to examine whether there is a relation between diet diversity and population dynamics.

## AUTHOR CONTRIBUTIONS

**Magne Neby:** Conceptualization; data curation; formal analysis; funding acquisition; investigation; methodology; project administration; resources; visualization; writing – original draft; writing – review and editing. **Rolf A. Ims:** Conceptualization; investigation; methodology; writing – review and editing. **Stefaniya Kamenova:** Conceptualization; methodology; resources; writing – review and editing. **Olivier Devineau:** Conceptualization; methodology; writing – review and editing. **Eeva M. Soininen:** Conceptualization; investigation; methodology; writing – review and editing.

## FUNDING INFORMATION

This study was funded by the Inland Norway University of Applied Sciences and the Royal Norwegian Society of Sciences and Letters (awarded April 23rd 2020). SK received funding from the EU Horizon 2020 Programme for Research and Innovation through Action Number 869471 (CHARTER: Drivers and Feedbacks of Changes in Arctic Terrestrial Biodiversity, PI: Bruce Forbes) as well as the Research Council of Norway‐funded project 315454 (PRISM: Understanding climate change impacts in an Arctic ecosystem: an integrated approach through the prism of Svalbard reindeer, PI: Leif Egil Loe). The funders had no role in the study design, data collection and analysis, or the preparation of and decision to publish the manuscript.

## CONFLICT OF INTEREST STATEMENT

The authors declare that they have no conflict of interest.

## Supporting information


Appendix S1

Appendix S2

Appendix S3

Appendix S4

Appendix S5

Appendix S6

## Data Availability

Raw read sequencing data reported in this paper have been deposited in the European Nucleotide Archive (ENA) under accession number PRJEB43470 and can be publicly accessed at https://www.ebi.ac.uk/ena/. The filtered data set and a guide for demultiplexing the raw files are available at https://gitlab.com/rodent/diet/. Further description of commands in the ObiTools package (Boyer et al., 2016) is found at: https://pythonhosted.org/OBITools/.
