# Supplementary material for: Is the diet cyclic phase‐dependent in boreal vole populations?
Source: Ecol Evol. 2024 Apr 17;14(4):e11227. doi: 10.1002/ece3.11227 (PMC11024456; doi:10.1002/ece3.11227)
Supplement: Supplementary file 1 — Appendix S1 [file ECE3-14-e11227-s001.docx]

**Supporting Information 1 – Supplementary information about the study area**

**Article name:** Is the diet cyclic phase-dependent in boreal vole populations?

**Journal name:** Ecology and Evolution, submitted.

**Author names:** Magne Neby^1,2*^, Rolf A. Ims^3^, Stefaniya Kamenova^4,5^, Olivier Devineau^1^, Eeva M. Soininen^3^

^1^ Department of Applied Ecology, Inland Norway University of Applied Sciences, Koppang, Norway

^2^ Department of Agricultural Sciences, Inland Norway University of Applied Sciences, Hamar, Norway

^3^ Department of Arctic and Marine Biology, UiT – the Arctic University of Norway, Tromsø, Norway

^4^Centre for Ecological and Evolutionary Synthesis, Department of Biosciences, University of Oslo, 0316 Oslo, Norway

^5^Faculty of Environmental Sciences and Natural Resource Management, Norwegian University of Life Sciences, 1432 Ås, Norway

***Corresponding author:**

Magne Neby

Department of Agricultural Sciences, Inland Norway University of Applied Sciences, Høyvangvegen 40, 2322 Ridabu, Norway.

Email address: [magne.neby@inn.no](mailto:magne.neby@inn.no)

**Weather conditions in the study area**

The climate is relatively continental (Boonstra et al. 2016), with a mean annual air temperature of 3°C and precipitation 571 mm (based on measurements at the Evenstad weather station during 1974-2019 (MET Norway 2021)). During this study, snow melted in May, and permanent snow cover commenced between October and November (Table ESM1.1). The mean daily temperatures were above 10 degrees C from April/May, and below in September (Table ESM1.1).

**Food availability in the study area**

We aimed to describe the most commonly available vegetation, lichens and fungi potentially used as food by voles. Vegetation and lichens were sampled mainly during plant peak biomass each year (see dates in Table ESM1.2), and survey methods were adapted to each type of habitat. In the forest, we used a 40 x 40 cm point-frame quadrat with nine evenly distributed pins (ø=4mm) and recorded every contact of a species/plant group by each of the pins (Lid et al. 2005; Mossberg & Stenberg 2003). This quadrat was used on two randomly selected vole trapping grids on 16 randomly selected plots (i.e., coordinates generated randomly within the grid), resulting in 288 pins. The forest’s dominance of spruce (and partly pine) in the canopy is not well reflected in the food availability data due to only measuring ground level, however, we registered seedlings and young tree plants. This point frame was impractical to use in the field habitat dominated by tall plants and was thus replaced with the bamboo sticks used for locating the traps (ø=16mm), thus resulting in only one pin per plot, and a total 108 pins for this habitat. We adapted the point-frame method to fit each habitat’s vegetation structure thus the vegetation compositions are only comparable qualitatively. In addition, all vegetation, lichens, and mushroom species/groups occurring at the trapping sites were noted as observed or not observed (Bacon & Sterry 2019; Holien & Tønsberg 2008). The point-frame method and the visual estimates provided similar data thus, the latter was not included further.

We observed contrasts in functional types, growth forms and in the most common species between the forest and field habitat (Figure 1 in main text). A complete list of the vegetation observed during systematic sampling, as well as all vegetation and fungi observed outside the sampling plots but inside the vole trapping areas (present/absent) are found in Table ESM1.2 and Table ESM1.3 below. Considering the difference in the sampling method in order to fit each habitat’s vegetation structure, the datasets are not directly comparable. Overall, we observe a higher diversity in the field habitat than in the forest habitat (Figure 1 in main text). All habitats in this biome have seasonal variation in the vegetation, but the field habitat more than the forest due to having the largest proportions of forbs and deciduous shrubs/trees (Figure ESM1.1 below).

| **Table ESM1.1. Seasons were defined based on snow cover.** Temperature data from Met Norway (2021) and snow cover from the lowermost trapping area. We defined the first day of the summer season when the snow becomes rare at the trapping grids. The summer lasted until winter which was defined as the date with permanent snow cover (i.e., the snow did not melt away afterwards), either after multiple short-term snowfall^1^ or after first snowfall^2^. | | | |
| --- | --- | --- | --- |
|  | **2017** | **2018** | **2019** |
| **Snow became rare at dates** | 19-May-2017 | 11-May-2018 | 01-May-2019 |
| **Permanent snow cover from** | 25-Oct-2017^1^ | 20-Nov-2018^2^ | 22-Nov-2019^2^ |
| **Daily temperatures > 10° C** | 05-May- 2017 | 05-May-2018 | 27-April-2019 |
| **Daily temperatures < 10° C** | 27-Sep-2017 | 20-Sep-2018 | 20-Sep-2019 |

| **Table ESM1.2.** Overview of vegetation (including bryophytes and lichens) observed during 13-20 July 2017, 14-21 July 2018 and 2-10 July 2019. The vegetation was monitored annually by visual estimates and point-frame methodology sampled randomly on the trapping grids/transects. The asterix denote when the species was observed in the grid/transect but not during sampling. | | |
| --- | --- | --- |
| **Scientific name** | **Observed in habitat** | **Observed in DNA** |
| *Achillea millefolium* | Forest | NA |
| *Aconitum lycoctonum* | Forest | NA |
| *Agrostis canina* | Forest | NA |
| *Agrostis capillaris* | Forest & Field habitat* | NA |
| *Agrostis gigantea* | Field habitat* | NA |
| *Alchemilla* sp. | Forest | NA |
| *Alchemilla vulgaris* | Forest | NA |
| *Allium oreophilum* | Forest | NA |
| *Alnus incana* | Forest | *Alnus* sp./spp. |
| *Alopecurus pratensis pratensis* | Field habitat* | NA |
| *Amblystegium serpens* | Field habitat* | NA |
| *Angelica sylvestris* | Field habitat* | NA |
| *Anthoxanthum odoratum* | Forest | NA |
| *Anthriscus sylvestris* | Forest & Field habitat* | *Anthriscus sylvestris* |
| *Athyrium filix-foemina* | Forest & Field habitat* | NA |
| *Barbilophozia* sp. | Field habitat | NA |
| *Betula* sp. | Forest & Field habitat | *Betula* sp./spp. |
| *Betula pendula* | Forest & Field habitat* | *Betula* sp./spp. |
| *Betula pubescens* | Forest & Field habitat* | *Betula* sp./spp. |
| *Bistorta vivipara* | Forest | NA |
| *Bromus* sp. | Field habitat | NA |
| *Bromus inermis* | Field habitat* | *Bromus inermis* |
| *Bryopsida* sp. | Field habitat* | NA |
| *Bryoria capillaris* | Forest | NA |
| *Bryoria fuscescens* | Forest | NA |
| *Calamagrostis* sp. | Forest & Field habitat | *Calamagrostis* sp./spp. |
| *Calamagrostis phragmitoides* | Forest & Field habitat* | *Calamagrostis* sp./spp. |
| *Calamagrostis purpurea* | Forest & Field habitat* | *Calamagrostis* sp./spp. |
| *Callitriche verna* | Field habitat* | NA |
| *Calluna* sp. | Field habitat | NA |
| *Calluna vulgaris* | Forest | *Calluna vulgaris* |
| *Caltha palustris* | Field habitat* | *Caltha* sp./spp. |
| *Cardamine amara* | Field habitat* | NA |
| *Carex* sp. | Forest & Field habitat* | NA |
| *Carex brunnescens* | Forest | NA |
| *Carex canescens* | Forest | NA |
| *Cerastium fontanum* | Forest | NA |
| *Chamerion* sp. | Field habitat | NA |
| *Chamerion angustifolium* | Forest & Field habitat* | *Chamerion angustifolium* |
| *Chenopodium album* | Forest & Field habitat* | NA |
| *Cicerbita alpina* | Forest | NA |
| *Cicuta virosa* | Forest & Field habitat* | NA |
| *Cirsium arvense* | Field habitat* | NA |
| *Cladonia* sp. | Forest & Field habitat | NA |
| *Cladonia arbuscula* | Forest | NA |
| *Cladonia bellidiflora* | Forest | NA |
| *Cladonia gracilis* | Forest | NA |
| *Cladonia rangiferina* | Forest | NA |
| *Cladonia stellaris* | Forest | NA |
| *Comarum palustre* | Field habitat* | *Comarum palustre* |
| *Convallaria majalis* | Forest | NA |
| *Crepis paludosa* | Forest | NA |
| *Cystopteris* sp. | Field habitat* | NA |
| *Dactylorhiza fuchsii* | Forest | NA |
| *Dactylorhiza maculata* | Forest | NA |
| *Deschampsia* sp. | Field habitat | NA |
| *Deschampsia cespitosa* | Forest & Field habitat* | NA |
| *Deschampsia flexuosa* | Forest | NA |
| *Dicranum* sp. | Forest & Field habitat | NA |
| *Dicranum scoparium* | Forest | NA |
| *Diphasiastrum complanatum* | Forest | NA |
| *Dryopteris* sp. | Field habitat | NA |
| *Dryopteris carthusiana* | Field habitat* | NA |
| *Dryopteris expansa* | Forest | NA |
| *Elymus* sp. | Field habitat | NA |
| *Elymus repens* | Field habitat* | NA |
| *Empetrum* sp. | Field habitat | *Empetrum* sp./spp. |
| *Empetrum nigrum* | Forest | *Empetrum* sp./spp. |
| *Epilobium* sp. | Forest & Field habitat | *Epilobium* sp./spp. |
| *Equisetum* sp. | Field habitat | NA |
| *Equisetum arvense* | Forest & Field habitat* | NA |
| *Equisetum fluviatile* | Forest | NA |
| *Equisetum sylvaticum* | Forest & Field habitat* | NA |
| *Erysimum cheiranthoides* | Field habitat* | NA |
| *Exobasidium* sp. | Forest | NA |
| *Festuca ovina* | Forest | *Festuca* sp./spp. |
| *Filipendula* sp. | Field habitat | NA |
| *Filipendula ulmaria* | Forest & Field habitat* | *Filipendula ulmaria* |
| *Fragaria vesca* | Forest | NA |
| *Galeopsis* sp. | Field habitat | NA |
| *Galeopsis speciosa* | Forest & Field habitat* | NA |
| *Galeopsis tetrahit* | Forest & Field habitat* | NA |
| *Galium album* | Field habitat* | *Galium* sp./spp. |
| *Galium boreale* | Field habitat* | *Galium* sp./spp. |
| *Galium palustre* | Forest & Field habitat* | *Galium* sp./spp. |
| *Galium uliginosum* | Field habitat* | *Galium* sp./spp. |
| *Geranium sylvaticum* | Forest & Field habitat* | NA |
| *Geum rivale* | Forest & Field habitat* | *Geum* sp./spp. |
| *Gnaphalium norvegicum* | Forest | NA |
| *Gymnocarpium* sp. | Field habitat | NA |
| *Gymnocarpium dryopteris* | Forest & Field habitat* | NA |
| *Hieracium* sp. | Forest | NA |
| *Hieracium murorum* | Forest | NA |
| *Hordeum* sp. | Field habitat* | *Hordeum* sp./spp. |
| *Hylocomium* sp. | Field habitat | NA |
| *Hylocomium splendens* | Forest | NA |
| *Hypogymnia* sp. | Forest | NA |
| *Hypogymnia physodes* | Forest & Field habitat* | NA |
| *Imshaugia aleurites* | Forest | NA |
| *Juniperus communis* | Forest | *Juniperus* sp./spp. |
| *Lathyrus pratensis* | Forest & Field habitat* | *Lathyrus pratensis* |
| *Leccinum versipelle* | Forest | NA |
| *Leucanthemum vulgare* | Forest | NA |
| *Linnaea borealis* | Forest | *Linnaea borealis* |
| *Linnea* sp. | Field habitat | NA |
| *Luzula multiflora* | Forest | NA |
| *Luzula pilosa* | Forest | *Luzula pilosa* |
| *Lycopodium annotinum* | Forest | NA |
| *Lycopodium clavatum* | Forest | NA |
| *Lysimachia europaea* | Forest & Field habitat* | NA |
| *Maianthemum* sp. | Field habitat | *Maianthemum* sp./spp. |
| *Maianthemum bifolium* | Forest & Field habitat* | *Maianthemum* sp./spp. |
| *Matricaria discoidea* | Forest & Field habitat* | NA |
| *Melampyrum* sp. | Field habitat | NA |
| *Melampyrum pratense* | Forest & Field habitat* | *Melampyrum pratense* |
| *Melampyrum sylvaticum* | Forest | *Melampyrum sylvaticum* |
| *Melica nutans* | Forest | NA |
| *Milium effusum* | Forest | NA |
| *Myosotis sylvatica* | Forest | NA |
| *Neottia ovata* | Forest | NA |
| *Oxalis acetocella* | Forest | NA |
| *Paris quadrifolia* | Forest & Field habitat* | NA |
| *Parmelia sulcata* | Forest & Field habitat* | NA |
| *Parmeliopsis ambigua* | Forest | NA |
| *Parmeliopsis hyperopta* | Forest | NA |
| *Persicaria lapathifolia* | Field habitat* | NA |
| *Phalaris arundinacea* | Field habitat* | NA |
| *Phegopteris* sp. | Field habitat | NA |
| *Phegopteris connectilis* | Forest & Field habitat* | NA |
| *Phleum* sp. | Field habitat | NA |
| *Phleum alpinum* | Forest | NA |
| *Phleum pratense* | Forest & Field habitat* | NA |
| *Picea* sp. | Field habitat | *Picea* sp./spp. |
| *Picea abies* | Forest | *Picea* sp./spp. |
| *Pinus* sp. | Field habitat | *Pinus* sp./spp. |
| *Pinus sylvestris* | Forest | *Pinus* sp./spp. |
| *Plantago major* | Forest & Field habitat* | *Plantago* sp./spp. |
| *Platismatia* sp. | Forest | NA |
| *Pleurozium* sp. | Field habitat | NA |
| *Pleurozium schreberi* | Forest & Field habitat* | NA |
| *Poa* sp. | Field habitat | *Poa* sp./spp. |
| *Poa annua* | Forest & Field habitat* | *Poa* sp./spp. |
| *Poa pratensis* | Forest & Field habitat* | *Poa* sp./spp. |
| *Poa trivialis* | Forest | *Poa* sp./spp. |
| *Poaceae* | Field habitat | NA |
| *Polygonum* sp. | Forest & Field habitat* | *Polygonum* sp./spp. |
| *Polytrichum* sp. | Forest & Field habitat | NA |
| *Polytrichum commune* | Forest | NA |
| *Populus tremula* | Forest | *Populus* sp./spp. |
| *Prunella vulgaris* | Forest | NA |
| *Prunus* sp. | Field habitat | *Prunus* sp./spp. |
| *Prunus padus* | Forest & Field habitat* | *Prunus* sp./spp. |
| *Pseudevernia* sp. | Field habitat | NA |
| *Pseudevernia furfuracea* | Forest | NA |
| *Ptilium* sp. | Field habitat | NA |
| *Ptilium crista-castrensis* | Forest | NA |
| *Ranunculus* sp. | Forest & Field habitat | *Ranunculus* sp./spp. |
| *Ranunculus acris* | Forest | *Ranunculus* sp./spp. |
| *Ranunculus repens* | Field habitat* | *Ranunculus* sp./spp. |
| *Rhinanthus minor* | Forest | NA |
| *Rhodobryum roseum* | Forest & Field habitat* | NA |
| *Ribes* sp. | Forest & Field habitat* | *Ribes* sp./spp. |
| *Rorippa sylvestris* | Field habitat* | NA |
| *Rosa* sp. | Field habitat | NA |
| *Rosa villosa* | Field habitat* | NA |
| *Rubus* sp. | Field habitat | *Rubus* sp./spp. |
| *Rubus idaeus* | Forest & Field habitat* | *Rubus* sp./spp. |
| *Rubus saxatilis* | Forest | *Rubus* sp./spp. |
| *Rumex* sp. | Forest & Field habitat* | *Rumex* sp./spp. |
| *Rumex acetosa* | Forest & Field habitat* | *Rumex* sp./spp. |
| *Rumex longifolius* | Field habitat* | *Rumex* sp./spp. |
| *Salix* sp. | Field habitat | NA |
| *Salix caprea* | Forest & Field habitat* | NA |
| *Salix cinerea* | Field habitat* | NA |
| *Salix myrsinifolia* | Forest & Field habitat* | NA |
| *Salix pentandra* | Forest | NA |
| *Salix starkeana* | Forest | NA |
| *Sambucus racemosa* | Field habitat* | *Sambucus* sp./spp. |
| *Scirpus sylvaticus* | Forest & Field habitat* | NA |
| *Scorzoneroides autumnalis* | Forest | NA |
| *Silene dioica* | Forest | *Silene dioica* |
| *Sinapis* sp. | Field habitat* | NA |
| *Solidago virgaurea* | Forest | NA |
| *Sorbus aucuparia* | Forest & Field habitat* | NA |
| *Spergula* sp. | Forest & Field habitat* | NA |
| *Sphagnum* sp. | Forest & Field habitat | NA |
| *Sphagnum girgensohnii* | Forest | NA |
| *Stellaria graminea* | Forest | *Stellaria* sp./spp. |
| *Stellaria media* | Forest & Field habitat* | *Stellaria* sp./spp. |
| *Stereocaulon paschale* | Forest | NA |
| *Tanacetum* sp. | Field habitat | NA |
| *Tanacetum vulgare* | Forest & Field habitat* | NA |
| *Taraxacum* sp. | Field habitat* | NA |
| *Trichophorum alpinum* | Forest | NA |
| *Trientalis europaea* | Forest | *Trientalis* sp./spp. |
| *Trifolium hybridum* | Forest & Field habitat* | NA |
| *Trifolium medium* | Forest | NA |
| *Trifolium pratense* | Forest & Field habitat* | NA |
| *Trifolium repens* | Forest & Field habitat* | *Trifolium repens* |
| *Tritomaria* sp. | Field habitat | NA |
| *Tritomaria quinquedentata* | Forest | NA |
| *Tussilago farfara* | Forest | NA |
| *Urtica* sp. | Field habitat | *Urtica* sp./spp. |
| *Urtica dioica* | Forest & Field habitat* | *Urtica* sp./spp. |
| *Usnea filipendula* | Forest | NA |
| *Usnea subfloridana* | Forest | NA |
| *Vaccinium* sp. | Field habitat | *Vaccinium* sp./spp. |
| *Vaccinium myrtillus* | Forest | *Vaccinium* sp./spp. |
| *Vaccinium uliginosum* | Forest | *Vaccinium* sp./spp. |
| *Vaccinium vitis-idaea* | Forest | *Vaccinium vitis-idaea* |
| *Valeriana sambucifolia* | Forest & Field habitat* | *Valeriana* sp./spp. |
| *Veronica* sp. | Forest | NA |
| *Vicia* sp. | Field habitat | NA |
| *Vicia cracca* | Forest & Field habitat* | *Vicia cracca* |
| *Viola lutea* | Field habitat* | NA |
| *Viola palustris* | Field habitat* | NA |
| *Viola tricolor* | Field habitat* | NA |
| *Viola uliginosa* | Forest | NA |
| *Vulpicida pinastri* | Forest* | NA |

| **Table ESM1.3.** Mushrooms observed during 2019 in the study area. |
| --- |
| *Agrocybe pediades* |
| *Amanita muscaria* |
| *Amanita pantherina* |
| *Auricularia auricula-judae* |
| *Boletus badius* |
| *Boletus* sp. |
| *Calocera viscosa* |
| *Clitocybe* sp. / spp. |
| *Clytocybe rivulosa* |
| *Cortinarius alboviolaceus* |
| *Cortinarius armillatus* |
| *Cortinarius sanguineus* |
| *Cortinarius* sp. / spp. |
| *Craterellus tubaeformis* |
| *Cystoderma* sp. |
| *Flammulina* sp. |
| *Gloeophyllum odoratum* |
| *Gymnopus* sp. |
| *Hydnum rufescens* |
| *Hygrophorus hypothejus* |
| *Hypholoma* sp. |
| *Lactarius rufus* |
| *Lactarius* sp. / spp. |
| *Lactarius torminosus* |
| *Lactarius vellereus* |
| *Lactarius vietus* |
| *Leccinium scarbrum* |
| *Leccinium vercipelle* |
| *Leccinum* sp. |
| *Lichenomphalia umbellifera* |
| *Marasmius rotula* |
| *Mycena galopus var. candida* |
| *Mycena* sp. / spp. |
| *Paxillus atrotomentosus* |
| *Psathyrella candolleana* |
| *Russula* sp. / spp. |
| *Strobilurus* sp. |
| *Suillus* sp. |
| *Suillus variegatus* |
| *Tricholoma terreum* |


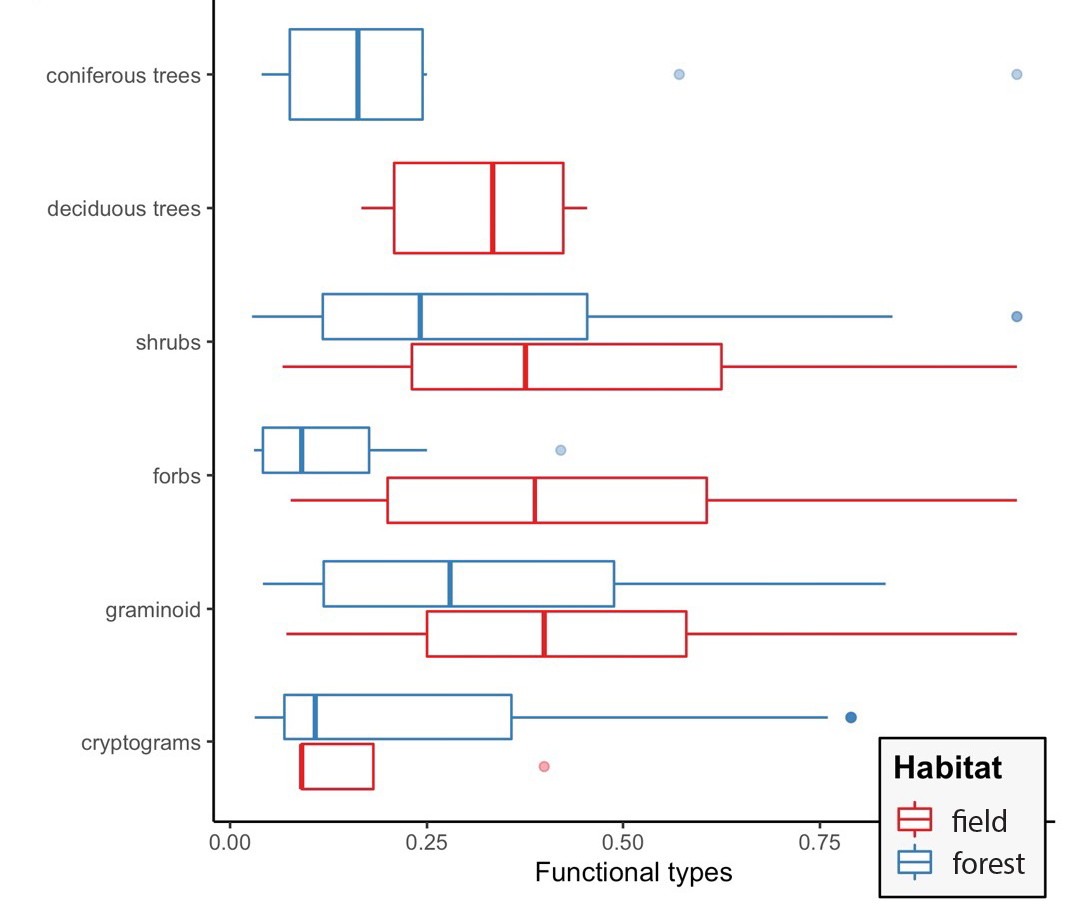


**Figure ESM1.1.** Characteristics of the vegetation based on point-frame data in the study area, classified according to the plant functional type.
