# Supplementary material for: Is the diet cyclic phase‐dependent in boreal vole populations?
Source: Ecol Evol. 2024 Apr 17;14(4):e11227. doi: 10.1002/ece3.11227 (PMC11024456; doi:10.1002/ece3.11227)
Supplement: Supplementary file 2 — Appendix S2 [file ECE3-14-e11227-s003.docx]

**Online Resource 2 – Supplementary information comparing faeces sampling methods**

**Article name:** Is the diet cyclic phase-dependent in boreal vole populations?

**Journal name:** Unpublished manuscript

**Author names:** Magne Neby^1,2*^, Rolf A. Ims^3^, Stefaniya Kamenova^4,5^, Olivier Devineau^1^, Eeva M. Soininen^3^

^1^ Department of Applied Ecology, Inland Norway University of Applied Sciences, Koppang, Norway

^2^ Department of Agricultural Sciences, Inland Norway University of Applied Sciences, Hamar, Norway

^3^ Department of Arctic and Marine Biology, UiT – the Arctic University of Norway, Tromsø, Norway

^4^Centre for Ecological and Evolutionary Synthesis, Department of Biosciences, University of Oslo, 0316 Oslo, Norway

^5^Faculty of Environmental Sciences and Natural Resource Management, Norwegian University of Life Sciences, 1432 Ås, Norway

***Corresponding author:**

Magne Neby

Department of Agricultural Sciences, Inland Norway University of Applied Sciences, Høyvangvegen 40, 2322 Ridabu, Norway.

Email address: [magne.neby@inn.no](mailto:magne.neby@inn.no)

Comparison between faeces sampling methods

We collected faecal samples in August and September 2017, and every month from January 2018 until August 2019 to analyse the diet of the voles. Whenever possible, we collected faeces directly from the traps during captures, selecting traps that contained >10 faecal pellets. However, since few voles were trapped at low vole densities, we also collected faeces (10 pellets) inside the traps when activating the traps before a primary trapping session. The trap's interior is relatively sheltered from the environment and stays relatively clean between primary trapping occasions. The voles use the inactive open traps in their runways, thus leaving faeces suitable for sample collection. Thus, if finding faeces before starting the trapping, such faeces stems from animals that had used the open traps in-between two primary sessions trapping (during the study, we observed that the defecation timing is distributed closer to deactivation of the former than activation of the successive session). Samples were considered as independent being deposited at least 48 hours since the direct method of sampling (Kostelecka-Myrcha and Myrcha 1964). However, the indirect means of sampling may potentially represent the diet of more than one individual. We thus assessed the correspondence between the two sampling approaches.

We validated the data pooling from the two faeces sampling methods (*directly* from the trap with captured vole individuals versus *indirectly* from an empty trap). We filtered out all the plant genera present in less than 5 % of the samples to reduce the number of zeros in the dataset. Followed by an imputation of zeros using impRZilr function in robComposition package (Templ et al. 2011). The plant genera that were detected in both types of samples (i.e. direct, indirect) were kept before estimating the coefficient of correlation using the acor function with alfa=1 (suggested after running acor.tune function) using the Compositional package (Tsagris et al. 2016). A threshold of 0.7 was set if the two types should be treated as one in the following analyses. In total, 143 samples were collected indirectly. Due to the uncertain species-identification associated with this method, these samples were subsequently Sanger sequenced, and 44 samples (31 %) were removed before further analysis due to misidentification (Suppl. Table C). Alpha generalised correlation (Tsagris et al. 2016) between two subsets of the plant dataset gave a very strong correlation between direct and indirect types of sampling for both tundra voles (0.84) and bank voles (0.94). Based on the coefficients being higher than our threshold of 0.7 we decided to treat the two types of sampling faeces as one, for all primer datasets and returned to the whole table for further analysis.

**References**

Kostelecka-Myrcha A, Myrcha A (1964) The rate of passage of foodstuffs through the alimentary tract of certain Microtidae under laboratory canditions. Acta Theriologica 9:37-53

Templ M, Hron K, Filzmoser P (2011) robCompositions: an R-package for robust statistical analysis of compositional data. Compositional Data Analysis: Theory and Applications. John Wiley and Sons, pp 341--355

Tsagris M, Preston S, Wood ATA (2016) Improved Classification for Compositional Data Using the α-transformation. Journal of Classification 33:243-261. doi: 10.1007/s00357-016-9207-5
