# Supplementary material for: Is the diet cyclic phase‐dependent in boreal vole populations?
Source: Ecol Evol. 2024 Apr 17;14(4):e11227. doi: 10.1002/ece3.11227 (PMC11024456; doi:10.1002/ece3.11227)
Supplement: Supplementary file 3 — Appendix S3 [file ECE3-14-e11227-s005.docx]

**Supporting Information 3 – Supplementary information on the performance of molecular analysis**

**Article name:** Is the diet cyclic phase-dependent in boreal vole populations?

**Journal name:** Ecology and Evolution

**Author names:** Magne Neby^1,2*^, Rolf A. Ims^3^, Stefaniya Kamenova^4,5,6^, Olivier Devineau^1^, Eeva M. Soininen^3^

^1^ Department of Applied Ecology, Inland Norway University of Applied Sciences, Koppang, Norway

^2^ Department of Agricultural Sciences, Inland Norway University of Applied Sciences, Hamar, Norway

^3^ Department of Arctic and Marine Biology, UiT – the Arctic University of Norway, Tromsø, Norway

^4^ Centre for Ecological and Evolutionary Synthesis, Department of Biosciences, University of Oslo, 0316 Oslo, Norway

^5^ Faculty of Environmental Sciences and Natural Resource Management, Norwegian University of Life Sciences, 1432 Ås, Norway

^6^ National Museum of Natural History, Bulgarian Academy of Sciences, Sofia, Bulgaria

***Corresponding author:**

Magne Neby

Department of Agricultural Sciences, Inland Norway University of Applied Sciences, Høyvangvegen 40, 2322 Ridabu, Norway.

Email address: [magne.neby@inn.no](mailto:magne.neby@inn.no)

**Sanger sequencing**

We validated the taxonomic identity of unidentified faecal samples using the set of arvicoline-specific primers Pro+/MicoMico (Alasaad et al. 2011), targeting the mitochondrial DNA control region. These are specifically designed to identify arvicoline mammals based on field-collected faecal samples (Verkuil et al. 2018). We used the same DNA extractions as described below. Forward Sanger sequences were analysed first in order to minimize sequencing costs, and the Reverse direction was sequenced only when The Basic Local Alignment Search Tool (BLAST) searches provided uncertain species identities. PCR products were cleaned using a combination of Illustra Exonuclease I and Alkaline Phosphatase with a first incubation at 37^o^C for 15 minutes, followed by a second incubation at 80^o^C for 15 minutes. Sanger sequencing was carried out at Eurofins Genomics, Belgium.

| Table ESM3.1. Overview of the six synthetic standard sequences used as PCR positive control mock community with the seed plants *Sper01* primers. Note that only standard 1 was used as a PCR positive control with the bryophytes, fungi, and eukaryotes primers. | | | | | | | |
| --- | --- | --- | --- | --- | --- | --- | --- |
| **Standard** | **Sequence (5’-3’)** | | | **Size (bp)** | **GC content (%)** | **Dilution factor** | |
| 1 | taagtctcgcactagttgtgacctaacgaatagagaattctataagacgtgttgtcccat | | | 60 | 40 | 1 | |
| 2 | gtgtatggtatatttgaataatattaaatagaatttaatcaatctttacatcgcttaata | | | 60 | 20 | 0.5 | |
| 3 | cacaatgctcggtaactagaagcatttgta | | | 30 | 40 | 0.25 | |
| 4 | attgaatgaaaagattattcgatatagaat | | | 30 | 20 | 0.125 | |
| 5 | agaacgctagaatctaagatggggggggggatgagtaagatatttatcagtaacatatga | | | 60 | 40 | 0.0652 | |
| 6 | atttttgtaactcattaacaattttttttttgatgtatcataagtactaaactagttact | | | 60 | 20 | 0.03125 | |
| Table ESM3.2. Pipeline and statistics using the ObiTools protocol. The sequencing batch number the two libraries from each primer pair respectively. The ObiTools commands correspond to the flowchart in Figure 4. GH = *Sper01* primers (seed plants), EUKA = *Euka02* primers (eukaryotes), FUNGI = *Fung01* primers (Fungi), BRYO = *Bryo01* primers (bryophytes), ZEALE = *ZBJ-ArtF1c/ ZBJ-ArtR2c* primers (arthropods). | | | | | | | |
| **OBITools command** | | **Sequencing batch** | **Number of reads** | | | | **Number of sequences** |
|  |  |  |  |  |  |  |  |
|  | |  | **Raw reads** (Sequencing run 1 / run 2) | | | |  |
|  | |  | All forward and reverse reads were the same length | | | |  |
|  | | GH 1 | 8030428 / 7179292 | | | | NA |
|  | | EUKA 1 | 6092336 / 5882105 | | | | NA |
|  | | FUNGI 1 | 5323198 / 11903499 | | | | NA |
|  | | GH 2* | 10929717 / 18998416 | | | | NA |
|  | | BRYO* | 10929717 / 18998416 | | | | NA |
|  | | EUKA 2 | 4610735 / 9198997 | | | | NA |
|  | | GH 3 | 3822973 / 6722859 | | | | NA |
|  | | EUKA 3 | 3931023 / 7619246 | | | | NA |
|  | | FUNGI 2 | 4307291 / 9821752 | | | | NA |
|  | | ZEALE 1 | 3010617 / 6554827 | | | | NA |
|  | | ZEALE 2 | 2113796 / 4291510 | | | | NA |
| *illuminapairedend* | | | **Alignment** | | | |  |
|  | |  | Recover full sequence reads from forward and reverse partial reads: Assembling pair-end reads. Discarded if overlapping quality was <40 | | | | |
|  | | GH 1 | 4015214 / 7179292 | | | | NA |
|  | | EUKA 1 | 3046168 / 5882105 | | | | NA |
|  | | FUNGI 1 | 5323198 / 11903499 | | | | NA |
|  | | GH 2* | 10929717 / 18998416 | | | | NA |
|  | | BRYO* | 10929717 / 18998416 | | | | NA |
|  | | EUKA 2 | 4610735 / 9198997 | | | | NA |
|  | | GH 3 | 3822973 / 6722859 | | | | NA |
|  | | EUKA 3 | 3931023 / 7619246 | | | | NA |
|  | | FUNGI 2 | 4307291 / 9821752 | | | | NA |
|  | | ZEALE 1 | 3010617 / 6554827 | | | | NA |
|  | | ZEALE 2 | 2113796 / 4291510 | | | | NA |
| *obigrep* | |  | **Filter sequences** | | | |  |
|  | |  | When the two reads overlap with a high alignment quality score, the consensus sequence is produced. Removes badly aligned sequences | | | | |
|  | | GH 1 | 4010624 / 7171499 | | | | NA |
|  | | EUKA 1 | 3023858 / 5847034 | | | | NA |
|  | | FUNGI 1 | 4259365 / 9387159 | | | | NA |
|  | | GH 2* | 10908511 / 18957285 | | | | NA |
|  | | BRYO* | 10908511 / 18957285 | | | | NA |
|  | | EUKA 2 | 4589968 / 9158781 | | | | NA |
|  | | GH 3 | 3777234 / 6653526 | | | | NA |
|  | | EUKA 3 | 3909477 / 7585603 | | | | NA |
|  | | FUNGI 2 | 3682215 / 8300434 | | | | NA |
|  | | ZEALE 1 | 2838662 / 6303598 | | | | NA |
|  | | ZEALE 2 | 1575285 / 3462983 | | | | NA |
| *ngsfilter* | |  | **Demultiplexing** | | | |  |
|  | |  | Assign each sequence to the corresponding sample | | | | |
|  | | GH 1 | 3331778 / 5928949 | | | | NA |
|  | | EUKA 1 | 2719274 / 5295462 | | | | NA |
|  | | FUNGI 1 | 3706656 / 8251993 | | | | NA |
|  | | GH 2 | 6066532 / 10526118 | | | | NA |
|  | | EUKA 2 | 4286861 / 6349779 | | | | NA |
|  | | GH 3 | 3404785 / 5969573 | | | | NA |
|  | | EUKA 3 | 3643649 / 7089267 | | | | NA |
|  | | BRYO | 3569213 / 6172646 | | | | NA |
|  | | FUNGI 2 | 3194724 / 4888533 | | | | NA |
|  | | ZEALE 1 | 2301354 / 5160713 | | | | NA |
|  | | ZEALE 2 | 1074959 / 2400423 | | | | NA |
| *obigrep* | |  | **Denoising** | | | |  |
|  | |  | Remove between-sample chimeras | | | |  |
|  | | GH 1 | 3088226 / 5482756 | | | | NA |
|  | | EUKA 1 | 2487035 / 4814748 | | | | NA |
|  | | FUNGI 1 | 3271742 / 7279193 | | | | NA |
|  | | GH 2 | 5630658 / 9742608 | | | | NA |
|  | | EUKA 2 | 3948226 / 5820462 | | | | NA |
|  | | GH 3 | 3127107 / 5468221 | | | | NA |
|  | | EUKA 3 | 3335162 / 6450940 | | | | NA |
|  | | BRYO | 3293190 / 5680022 | | | | NA |
|  | | FUNGI 2 | 2816491 / 4302368 | | | | NA |
|  | | ZEALE 1 | 2113242 / 4729548 | | | | NA |
|  | | ZEALE 2 | 964523 / 2151189 | | | | NA |
| *cat* | |  | **Merge libraries** | | | |  |
|  | |  | Merge sequencing runs and libraries based on the primer set | | | |  |
|  | | GH | 32539154 | | | | NA |
|  | | EUKA | 26856288 | | | | NA |
|  | | FUNGI | 17669794 | | | | NA |
|  | | BRYO | 8973212 | | | | NA |
|  | | ZEALE | 9958246 | | | | NA |
| *obiuniq* | |  | **Dereplication** | | | |  |
|  | |  | Group identical sequences into a unique sequence with count of sequence read per sample | | | |  |
|  | | GH | 32539154 | | | | 171916 |
|  | | EUKA | 26856288 | | | | 362638 |
|  | | FUNGI | 17669794 | | | | 1471301 |
|  | | BRYO | 8973212 | | | | 35192 |
|  | | ZEALE | 9958246 | | | | 475364 |
| *obigrep* | |  | **Remove singletons and short sequences** | | | |  |
|  | |  | Remove sequences with low frequency (<10) | | | | |
|  | | GH | 32275262 | | | | 13859 |
|  | | EUKA | 26299515 | | | | 29243 |
|  | | FUNGI | 15770091 | | | | 47807 |
|  | | BRYO | 8909449 | | | | 3766 |
|  | | ZEALE | 9296680 | | | | 21470 |
| *obiclean* | |  | **Cluster sequences and tag PCR errors** | | | |  |
|  | |  | Remove PCR and sequencing errors. Default distance threshold. | | | | |
|  | | GH | 32275262 | | | | 13859 |
|  | | EUKA | 26299515 | | | | 29243 |
|  | | FUNGI | 15770091 | | | | 47807 |
|  | | BRYO | 8909449 | | | | 3766 |
|  | | ZEALE | 9296680 | | | | 21470 |
| *ecotag* | |  | Assign head sequences to taxa. Criterion -m was not applied | | | |  |
|  | | GH | 32275262 | | | | 13859 |
|  | | EUKA | 26299515 | | | | 29243 |
|  | | FUNGI | 15770091 | | | | 47807 |
|  | | BRYO | 8909449 | | | | 3766 |
|  | | ZEALE | 9296680 | | | | 21470 |

| **Table ESM3.3. Details on filtering steps performed in R.** | | | | |  |
| --- | --- | --- | --- | --- | --- |
|  | **PCR replicates** | **samples** | | **MOTUs** | |
| GH | 600 | NA | | 1603 | |
| EUKA | 610 | NA | | 3669 | |
| FUNGI | 397 | NA | | 3701 | |
| BRYO | 189 | NA | | 183 | |
| ZEALE | 152 | NA | | 648 | |
| **Filter minimum frequency of MOTUs.** A lower threshold was used for EUKA and FUNGI primer datasets due to non-dietary coprophilous fungi dominating in the samples | | |  | |  |
| GH (1 %) | 600 | NA | | 150 | |
| EUKA (0.01 %) | 610 | NA | | 3221 | |
| FUNGI (0.01 %) | 397 | NA | | 1661 | |
| BRYO (1 %) | 189 | NA | | 51 | |
| ZEALE (1 %) | 152 | NA | | 173 | |
| **Removing tag jumps and possible contaminants using the positive control sequences** | |  | |  | |
| GH | 595 | NA | | 150 | |
| EUKA | 609 | NA | | 3221 | |
| FUNGI | 391 | NA | | 1661 | |
| BRYO | 188 | NA | | 51 | |
| ZEALE (DNA extracts from voucher specimens used instead of standard sequences) | | | | |  |
| **Remove PCR outliers** based on the comparison of Euclidean distances of PCR replicates with their average, and with the distribution of pairwise dissimilarities between all average samples. PCR replicates flagged as outliers were iteratively removed from the dataset. | |  | |  | |
| GH | 594 | NA | | 150 | |
| EUKA | 607 | NA | | 3221 | |
| FUNGI | 391 | NA | | 1661 | |
| BRYO | 188 | NA | | 51 | |
| ZEALE | 152 | NA | | 173 | |
| **Merge PCR replicates into samples** by averaging the number of reads per MOTU across the PCR replicates | |  | |  | |
| GH | NA | 202 | | 150 | |
| EUKA | NA | 204 | | 3221 | |
| FUNGI | NA | 200 | | 1661 | |
| BRYO | NA | 102 | | 51 | |
| ZEALE | NA | 92 | | 173 | |
| **Remove non-primer sequences and filter >0 read frequency** | | |  | |  |
| GH | NA | 202 | | 106 | |
| EUKA | NA | 204 | | 805 | |
| FUNGI | NA | 200 | | 82 | |
| BRYO | NA | 102 | | 42 | |
| ZEALE | NA | 92 | | 81 | |
| **Keep only taxonomic group aimed for** | | |  | |  |
| GH (only Spermatophyta) | NA | 202 | | 100 | |
| EUKA (only Eukaryota) | NA | 204 | | 772 | |
| FUNGI (only Fungi) | NA | NA | | NA | |
| BRYO (only Bryophyta) | NA | 102 | | 41 | |
| ZEALE (only Arthropoda) | NA | 92 | | 63 | |
| **Remove contaminants using DNA extraction and PCR negative controls** | |  | |  | |
| GH | NA | 202 | | 99 | |
| EUKA | NA | 202 | | 762 | |
| FUNGI | NA | 198 | | 81 | |
| BRYO | NA | 102 | | 41 | |
| ZEALE | NA | 90 | | 61 | |
| **Keep only faeces samples** (i.e., remove all the control samples) | |  | |  | |
| GH | NA | 196 | | 99 | |
| EUKA | NA | 200 | | 763 | |
| FUNGI | NA | 196 | | 81 | |
| BRYO | NA | 100 | | 41 | |
| ZEALE | NA | 89 | | 61 | |
| **Subset bank vole samples into a separate dataset** | |  | |  | |
| GH | NA | 128 | | 91 | |
| EUKA | NA | 131 | | 637 | |
| FUNGI | NA | 129 | | 55 | |
| BRYO | NA | 83 | | 35 | |
| ZEALE | NA | 73 | | 58 | |
| **Subset tundra vole samples into a separate dataset** | |  | |  | |
| GH | NA | 68 | | 90 | |
| EUKA | NA | 69 | | 551 | |
| FUNGI | NA | 67 | | 38 | |
| BRYO | NA | 17 | | 22 | |
| ZEALE | NA | 16 | | 16 | |
| **Other** |  |  | |  | |
| GH |  |  | |  | |
| - Remove MOTUs likely corresponding to the bait food used in the rodent traps | |  | |  | |
| - Forest | NA | 127 | | 86 | |
| - Field | NA | 66 | | 85 | |
| EUKA |  |  | |  | |
| - Remove non-lichen/non-mushroom fungal taxa and other non-dietary taxa based on known taxonomic groups | | | | |  |
| - Forest | NA | 131 | | 303 | |
| - Field | NA | 69 | | 247 | |
| FUNGI |  |  | |  | |
| - Subset Agaricomycetes and common lichen genera | | | | |  |
| - Forest | NA | 129 | | 4 | |
| - Field | NA | 0 | | 0 | |
|  | | | | |  |

**Table ESM3.4.** Sample size table showing the number of samples present after bioinformatics and data filtering, used for the statistical analyses. The number of samples originally collected are given inside parenthesis.

| **Year** | **Month** | **Field** | **Forest** |
| --- | --- | --- | --- |
| 2017 | Aug | 6 (6) | 7 (7) |
| - | Sep | 0 (0) | 6 (6) |
| 2018 | Jan | 7 (15) | 17 (18) |
| - | Feb | 1 (6) | 6 (6) |
| - | Mar | 7 (12) | 6 (6) |
| - | May | 6 (8) | 6 (6) |
| - | Jun | 10 (13) | 7 (7) |
| - | Jul | 12 (14) | 6 (6) |
| - | Aug | 12 (13) | 6 (6) |
| - | Sep | 3 (4) | 6 (6) |
| - | Oct | 1 (9) | 12 (12) |
| - | Nov | 0 (3) | 5 (5) |
| - | Dec | 1 (1) | 9 (11) |
| 2019 | Jan | 0 (0) | 7 (7) |
| - | Mar | 0 (0) | 5 (6) |
| - | Apr | 1 (1) | 2 (4) |
| - | May | 0 (1) | 3 (4) |
| - | Jun | 1 (1) | 3 (3) |
| - | Jul | 1 (1) | 6 (6) |
| - | Aug | 0 (0) | 6 (6) |

**References**

Alasaad S, Soriguer RC, Jowers MJ, Marchal JA, Romero I, Sánchez A (2011) Applicability of mitochondrial DNA for the identification of Arvicolid species from faecal samples: a case study from the threatened Cabrera’s vole. Mol Ecol Resour 11:409-414. doi: 10.1111/j.1755-0998.2010.02939.x

Verkuil YI, Van Guldener WEA, Lagendijk DDG, Smit C (2018) Molecular identification of temperate Cricetidae and Muridae rodent species using fecal samples collected in a natural habitat. Mammal Res. 63:379-385. doi: 10.1007/s13364-018-0359-z
