# Supplementary material for: Is the diet cyclic phase‐dependent in boreal vole populations?
Source: Ecol Evol. 2024 Apr 17;14(4):e11227. doi: 10.1002/ece3.11227 (PMC11024456; doi:10.1002/ece3.11227)
Supplement: Supplementary file 4 — Appendix S4 [file ECE3-14-e11227-s004.docx]

**Online Resource 4 – Supplementary information on the vole abundance estimates**

**Article name:** Is the diet cyclic phase-dependent in boreal vole populations?

**Journal name:** Unpublished manuscript

**Author names:** Magne Neby^1,2*^, Rolf A. Ims^3^, Stefaniya Kamenova^4,5^, Olivier Devineau^1^, Eeva M. Soininen^3^

^1^ Department of Applied Ecology, Inland Norway University of Applied Sciences, Koppang, Norway

^2^ Department of Agricultural Sciences, Inland Norway University of Applied Sciences, Hamar, Norway

^3^ Department of Arctic and Marine Biology, UiT – the Arctic University of Norway, Tromsø, Norway

^4^Centre for Ecological and Evolutionary Synthesis, Department of Biosciences, University of Oslo, 0316 Oslo, Norway

^5^Faculty of Environmental Sciences and Natural Resource Management, Norwegian University of Life Sciences, 1432 Ås, Norway

***Corresponding author:**

Magne Neby

Department of Agricultural Sciences, Inland Norway University of Applied Sciences, Høyvangvegen 40, 2322 Ridabu, Norway.

Email address: [magne.neby@inn.no](mailto:magne.neby@inn.no)

| **Models used for survival and abundance estimates on bank vole (A) and tundra vole (B) populations.** All other models yielded a ΔAIC >50. Model selection performed for parameters monthly survival rates and abundance of independent vole populations.. We used Robust Design with Closed Population Estimation model («Robust» in RMark). We selected the best (topmost in table) model. The model structure were survival (S), GammaDoublePrime (γ”), GammaPrime (γ’), probability of initial capture during a trapping session (p), probability of recapture during a trapping session conditional on initial capture (c). Time corresponds to the primary trapping sessions, grid corresponds to trapping grid/transect, session is primary trapping session and ~1 is constant. | | |
| --- | --- | --- |
| A) |  |  |
| **model** | **AICc** | **DeltaAICc** |
| S(~time)Gamma''(~1)Gamma'()p(~time)c()f0(~session) | 3327.6 | 0 |
| S(~time)Gamma''(~time)Gamma'()p(~time)c()f0(~session) | 3343.0 | 15.4 |
| S(~grid)Gamma''(~time)Gamma'()p(~time)c()f0(~session) | 3366.2 | 38.6 |
| S(~1)Gamma''(~time)Gamma'()p(~time)c()f0(~session) | 3367.0 | 39.4 |
|  |  |  |
| B) |  |  |
| **model** | **AICc** | **DeltaAICc** |
| S(~time)Gamma''(~1)Gamma'()p(~time)c()f0(~session) | 835.2 | 0 |
| S(~1)Gamma''(~1)Gamma'()p(~time)c()f0(~session) | 835.7 | 0.5 |
| S(~grid)Gamma''(~1)Gamma'()p(~time)c()f0(~session) | 837.9 | 2.7 |
| S(~1)Gamma''(~time)Gamma'()p(~time)c()f0(~session) | 841.2 | 5.9 |
| S(~grid)Gamma''(~time)Gamma'()p(~time)c()f0(~session) | 843.4 | 8.1 |
| S(~time)Gamma''(~time)Gamma'()p(~time)c()f0(~session) | 848.0 | 12.8 |
| S(~grid:time)Gamma''(~1)Gamma'()p(~time)c()f0(~session) | 851.2 | 16.0 |
| S(~grid:time)Gamma''(~time)Gamma'()p(~time)c()f0(~session) | 864.7 | 29.5 |
| S(~time)Gamma''(~1)Gamma'()p(~1)c()f0(~session) | 869.8 | 34.6 |
| S(~1)Gamma''(~1)Gamma'()p(~1)c()f0(~session) | 870.6 | 35.4 |
| S(~grid)Gamma''(~1)Gamma'()p(~1)c()f0(~session) | 872.7 | 37.5 |
| S(~1)Gamma''(~time)Gamma'()p(~1)c()f0(~session) | 875.8 | 40.6 |
| S(~grid)Gamma''(~time)Gamma'()p(~1)c()f0(~session) | 878.0 | 42.8 |
| S(~time)Gamma''(~time)Gamma'()p(~1)c()f0(~session) | 882.4 | 47.2 |
