# Supplementary material for: Is the diet cyclic phase‐dependent in boreal vole populations?
Source: Ecol Evol. 2024 Apr 17;14(4):e11227. doi: 10.1002/ece3.11227 (PMC11024456; doi:10.1002/ece3.11227)
Supplement: Supplementary file 5 — Appendix S5 [file ECE3-14-e11227-s002.docx]

**Supporting Information 5 – Supplementary information about the diet described by DNA barcoding**

**Article name:** Is the diet cyclic phase-dependent in boreal vole populations?

**Journal name:** Ecology and Evolution, submitted.

**Author names:** Magne Neby^1,2*^, Rolf A. Ims^3^, Stefaniya Kamenova^4,5^, Olivier Devineau^1^, Eeva M. Soininen^3^

^1^ Department of Applied Ecology, Inland Norway University of Applied Sciences, Koppang, Norway

^2^ Department of Agricultural Sciences, Inland Norway University of Applied Sciences, Hamar, Norway

^3^ Department of Arctic and Marine Biology, UiT – the Arctic University of Norway, Tromsø, Norway

^4^Centre for Ecological and Evolutionary Synthesis, Department of Biosciences, University of Oslo, 0316 Oslo, Norway

^5^Faculty of Environmental Sciences and Natural Resource Management, Norwegian University of Life Sciences, 1432 Ås, Norway

***Corresponding author:**

Magne Neby

Department of Agricultural Sciences, Inland Norway University of Applied Sciences, Høyvangvegen 40, 2322 Ridabu, Norway.

Email address: [magne.neby@inn.no](mailto:magne.neby@inn.no)

| a) Vascular plants detected in the faeces of tundra voles.  **Family (35)** | **Genus (59)** | **RRA (± SE)** | **wPOO** |
| --- | --- | --- | --- |
| Adoxaceae |  | 0.5 (±3.7) | 13.6 |
| " | *Sambucus* | 0.5 (±3.7) | 13.6 |
| Apiaceae |  | 2.9 (±0.7) | 92.4 |
| " | *Anthriscus* | 2.8 (±0.7) | 92.4 |
| " | *Cicuta* | 0 (±0.2) | 6.1 |
| Asteraceae |  | 0.6 (±0.4) | 59.1 |
| " | *Gnaphalium* | 0.6 (±0.4) | 59.1 |
| Betulaceae |  | 0.6 (±0.2) | 83.3 |
| " | *Alnus* | 0.1 (±0) | 56.1 |
| " | *Betula* | 0.5 (±0.2) | 75.8 |
| Boraginaceae |  | 0.1 (±0) | 1.5 |
| " | *Myosotis* | 0.1 (±0) | 1.5 |
| Brassicaceae |  | 0 (±0.2) | 3 |
| " | *Cakile* | 0 (±0.2) | 3 |
| Caprifoliaceae |  | 0 (±1) | 3 |
| " | *Valeriana* | 0 (±1) | 3 |
| Caryophyllaceae |  | 0.2 (±1.6) | 10.6 |
| " | *Silene* | 0.1 (±2.7) | 4.5 |
| " | *Stellaria* | 0 (±0.6) | 7.6 |
| Cupressaceae |  | 0 (±0) | 9.1 |
| " | *Juniperus* | 0 (±0) | 9.1 |
| Cystopteridaceae |  | 0 (±0) | 1.5 |
| " | *Gymnocarpium* | 0 (±0) | 1.5 |
| Dryopteridaceae |  | 0.1 (±1) | 7.6 |
| " | *Dryopteris* | 0.1 (±1) | 7.6 |
| Equisetaceae |  | 0 (±0) | 6.1 |
| " | *Equisetum* | 0 (±0) | 6.1 |
| Ericaceae |  | 0.3 (±1.3) | 16.7 |
| " | *Empetrum* | 0.2 (±0) | 1.5 |
| " | *Vaccinium* | 0.1 (±0.4) | 16.7 |
| Fabaceae |  | 0.3 (±0.3) | 45.5 |
| " | *Lathyrus* | 0.1 (±0.8) | 9.1 |
| " | *"* | 0.1 (±0.8) | 9.1 |
| " | *Trifolium* | 0 (±0.3) | 13.6 |
| " | *Vicia* | 0.2 (±0.2) | 37.9 |
| Grossulariaceae |  | 0.1 (±0.3) | 27.3 |
| " | *Ribes* | 0.1 (±0.3) | 27.3 |
| Juncaceae |  | 0 (±0) | 1.5 |
| " | *Juncus* | 0 (±0) | 1.5 |
| Lamiaceae |  | 0 (±0) | 1.5 |
| " | *Glechoma* | 0 (±0) | 1.5 |
| Menyanthaceae |  | 0.2 (±1) | 10.6 |
| " | *Menyanthes* | 0.2 (±1) | 10.6 |
| Onagraceae |  | 3.5 (±1.8) | 84.8 |
| " | *Chamaenerion* | 3.3 (±1.8) | 84.8 |
| " | *Epilobium* | 0.1 (±0.5) | 21.2 |
| Orobanchaceae |  | 0 (±0.2) | 6.1 |
| " | *Melampyrum* | 0 (±0.2) | 6.1 |
| Oxalidaceae |  | 0 (±0.1) | 3 |
| " | *Oxalis* | 0 (±0.1) | 3 |
| Pinaceae |  | 0.4 (±0.2) | 69.7 |
| " | *Picea* | 0.2 (±0.2) | 47 |
| " | *Pinus* | 0.1 (±0.1) | 56.1 |
| Plantaginaceae |  | 0 (±0.4) | 6.1 |
| " | *Plantago* | 0 (±0.4) | 6.1 |
| Poaceae |  | 26.2 (±2.9) | 100 |
| " | *Alopecurus* | 14.7 (±2.9) | 98.5 |
| " | *Agrostis* | 0.3 (±1.4) | 12.1 |
| " | *Avenella* | 0 (±0.2) | 7.6 |
| " | *Bromus* | 0 (±0.3) | 7.6 |
| " | *Calamagrostis* | 0 (±0) | 15.2 |
| " | *Festuca* | 0.3 (±0.9) | 21.2 |
| " | *Glyceria* | 0.1 (±0.6) | 4.5 |
| " | *Hordeum* | 9.6 (±1.9) | 89.4 |
| " | *Poa* | 1.2 (±1.1) | 53 |
| Polygonaceae |  | 1 (±1.5) | 42.4 |
| " | *Persicaria* | 0.2 (±1.1) | 12.1 |
| " | *Polygonum* | 0 (±0.2) | 9.1 |
| " | *Rumex* | 0.8 (±1.8) | 36.4 |
| Primulaceae |  | 0.2 (±1) | 16.7 |
| " | *Lysimachia* | 0.2 (±1.6) | 10.6 |
| " | *Trientalis* | 0 (±0.3) | 7.6 |
| Ranunculaceae |  | 3.1 (±1.6) | 75.8 |
| " | *Caltha* | 1.4 (±1.3) | 43.9 |
| " | *Ranunculus* | 1.8 (±1.9) | 59.1 |
| Rosaceae |  | 36.5 (±3.3) | 98.5 |
| " | *Comarum* | 0.3 (±0.4) | 65.2 |
| " | *Filipendula* | 13.2 (±2.4) | 87.9 |
| " | *Geum* | 0.2 (±4.7) | 4.5 |
| " | *Prunus* | 4 (±1.4) | 74.2 |
| " | *Rubus* | 18.9 (±2.9) | 93.9 |
| Rubiaceae |  | 0 (±0.2) | 16.7 |
| " | *Galium* | 0 (±0.2) | 16.7 |
| Rutaceae |  | 0 (±0) | 1.5 |
| " | *Choisya* | 0 (±0) | 1.5 |
| Salicaceae |  | 20.8 (±2.7) | 98.5 |
| " | *Populus* | 0 (±0) | 7.6 |
| " | *Salix* | 20.8 (±2.7) | 98.5 |
| Saxifragaceae |  | 0 (±0) | 3 |
| " | *Chrysosplenium* | 0 (±0) | 3 |
| Solanaceae |  | 0 (±0) | 6.1 |
| Thelypteridaceae |  | 0 (±0) | 7.6 |
| " | *Phegopteris* | 0 (±0) | 7.6 |
| Urticaceae |  | 2.2 (±1.4) | 75.8 |
| " | *Urtica* | 2.2 (±1.4) | 75.8 |
|  |  |  |  |
| Identified above family level | | 0 (±0.1) | 4.5 |

| b) Vascular plants detected in the faeces of bank voles.  **Family (35)** | **Genus (58)** | **RRA (± SE)** | | **wPOO** | |  |
| --- | --- | --- | --- | --- | --- | --- |
| Adoxaceae |  | 0 (±0) | | 12.6 | |  |
| " | *Sambucus* | 0 (±0) | | 12.6 | |  |
|  |  |  | |  | |  |
| Apiaceae |  | 15 (±2.3) | | 92.1 | |  |
| " | *Anthriscus* | 15 (±2.3) | | 92.1 | |  |
| Asparagaceae |  | 0 (±0.1) | | 16.5 | |  |
| " | *Maianthemum* | 0 (±0.1) | | 16.5 | |  |
|  |  |  | |  | |  |
| Asteraceae |  | 0 (±0.1) | | 12.6 | |  |
| " | *Gnaphalium* | 0 (±0.1) | | 12.6 | |  |
|  |  |  | |  | |  |
| Betulaceae |  | 11 (±1.9) | | 93.7 | |  |
| " | *Alnus* | 5.2 (±2.5) | | 46.5 | |  |
| " | *Betula* | 5.8 (±1.4) | | 90.6 | |  |
| Brassicaceae |  | 0.2 (±1.5) | | 11 | |  |
| " | *Cakile* | 0.2 (±1.5) | | 11 | |  |
|  |  |  | |  | |  |
| Caprifoliaceae |  | 0.7 (±0.6) | | 48.8 | |  |
| " | *Linnaea* | 0.7 (±0.6) | | 48.8 | |  |
| " | *Valeriana* | 0 (±0) | | 0.8 | |  |
| Caryophyllaceae |  | 0.1 (±0.3) | | 10.2 | |  |
| " | *Silene* | 0 (±0.2) | | 6.3 | |  |
| " | *Stellaria* | 0 (±0.3) | | 6.3 | |  |
| Cupressaceae |  | 0.1 (±0.2) | | 18.1 | |  |
| " | *Juniperus* | 0.1 (±0.2) | | 18.1 | |  |
| Cystopteridaceae |  | 0.2 (±0.8) | | 18.1 | |  |
| " | *Gymnocarpium* | 0.2 (±0.8) | | 18.1 | |  |
| Dryopteridaceae |  | 0 (±0) | | 9.4 | |  |
| " | *Dryopteris* | 0 (±0) | | 9.4 | |  |
| Equisetaceae |  | 0.1 (±0.3) | | 11.8 | |  |
| " | *Equisetum* | 0.1 (±0.3) | | 11.8 | |  |
| Ericaceae |  | 23.6 (±2.5) | | 98.4 | |  |
| " | *Calluna* | 2.2 (±6.8) | | 15 | |  |
| " | *Empetrum* | 1.2 (±4.6) | | 15 | |  |
| " | *Moneses* | 0.1 (±0.7) | | 7.1 | |  |
| " | *Vaccinium* | 20.2 (±2.3) | | 98.4 | |  |
| Fabaceae |  | 0 (±0) | | 6.3 | |  |
| " | *Trifolium* | 0 (±0.1) | | 2.4 | |  |
| " | *Vicia* | 0 (±0) | | 4.7 | |  |
| Grossulariaceae |  | 0 (±0.8) | | 3.9 | |  |
| " | *Ribes* | 0 (±0.8) | | 3.9 | |  |
| Juncaceae |  | 0.1 (±0.3) | | 14.2 | |  |
| " | *Juncus* | 0 (±0) | | 0.8 | |  |
| " | *Luzula* | 0.1 (±0.3) | | 13.4 | |  |
| Menyanthaceae |  | 0.2 (±1) | | 12.6 | |  |
| " | *Menyanthes* | 0.2 (±1) | | 12.6 | |  |
| Montiaceae |  | 0 (±0.1) | | 25.2 | |  |
| Onagraceae |  | 0.2 (±0.5) | | 27.6 | |  |
| " | *Chamaenerion* | 0.2 (±0.5) | | 26 | |  |
| " | *Epilobium* | 0 (±0) | | 1.6 | |  |
| Orobanchaceae |  | 9.8 (±1.9) | | 76.4 | |  |
| " | *Melampyrum* | 9.8 (±1.9) | | 76.4 | |  |
| Oxalidaceae |  | 1 (±2.2) | | 25.2 | |  |
| " | *Oxalis* | 1 (±2.2) | | 25.2 | |  |
| Pinaceae |  | 14.1 (±2.1) | | 100 | |  |
| " | *Picea* | 7.8 (±1.7) | | 92.9 | |  |
| " | *Pinus* | 6.3 (±1.5) | | 92.9 | |  |
| Plantaginaceae |  | 0 (±0) | | 0.8 | |  |
| " | *Plantago* | 0 (±0) | | 0.8 | |  |
| Poaceae |  | 14 (±2) | | 96.1 | |  |
| " | *Agrostis* | 0 (±0) | | 0.8 | |  |
| " | *Alopecurus* | 4.7 (±1.3) | | 85 | |  |
| " | *Avenella* | 5.2 (±2.5) | | 50.4 | |  |
| " | *Calamagrostis* | 0 (±1.4) | | 3.1 | |  |
| " | *Festuca* | 0.2 (±0.4) | | 23.6 | |  |
| " | *Hordeum* | 3.7 (±2.2) | | 43.3 | |  |
| " | *Muhlenbergia* | 0 (±0.2) | | 6.3 | |  |
| " | *Poa* | 0.2 (±0.2) | | 49.6 | |  |
| Polygonaceae |  | 0 (±0.1) | | 11.8 | |  |
| " | *Persicaria* | 0 (±0) | | 1.6 | |  |
| " | *Polygonum* | 0 (±0.1) | | 2.4 | |  |
| " | *Rumex* | 0 (±0.1) | | 11 | |  |
| Primulaceae |  | 0 (±0.1) | | 18.1 | |  |
| " | *Lysimachia* | 0 (±0.2) | | 3.1 | |  |
| " | *Trientalis* | 0 (±0.1) | | 15.7 | |  |
| Ranunculaceae |  | 0.2 (±0.3) | | 15.7 | |  |
| " | *Caltha* | 0 (±0) | | 4.7 | |  |
| " | *Ranunculus* | 0.2 (±0.4) | | 14.2 | |  |
| Rosaceae |  | 4.1 (±1.7) | | 57.5 | |  |
| " | *Comarum* | 0 (±0.2) | | 10.2 | |  |
| " | *Filipendula* | 1.5 (±1.7) | | 26 | |  |
| " | *Prunus* | 1.3 (±2.6) | | 19.7 | |  |
| " | *Rubus* | 1.2 (±1.1) | | 43.3 | |  |
| Rubiaceae |  | 0 (±0) | | 3.9 | |  |
| " | *Galium* | 0 (±0) | | 3.9 | |  |
| Rutaceae |  | 0 (±0.2) | | 1.6 | |  |
| " | *Choisya* | 0 (±0.2) | | 1.6 | |  |
| Salicaceae |  | 4.9 (±1.5) | | 66.9 | |  |
| " | *Populus* | 0.4 (±1) | | 19.7 | |  |
| " | *Salix* | 4.6 (±1.6) | | 64.6 | |  |
| Saxifragaceae |  | 0.1 (±1.6) | | 3.9 | |  |
| " | *Chrysosplenium* | 0.1 (±1.6) | | 3.9 | |  |
| Solanaceae |  | 0 (±0.2) | | 7.9 | |  |
| Thelypteridaceae |  | 0 (±0.4) | | 3.1 | |  |
| " | *Phegopteris* | 0 (±0.4) | | 3.1 | |  |
| Urticaceae |  | 0 (±0) | | 19.7 | |  |
| " | *Urtica* | 0 (±0) | | 19.7 | |  |
|  |  |  | |  | |  |
| Identified above family level | | | 0.1 (±1.1) | | 4.7 | |

**Taxonomic trees for group comparison independent of taxonomic resolution** (Zinger et al. 2021). The colours indicate mean relative read abundance (RRA) for each taxonomic level, similarly, the size of the points (%motus) indicate the frequency of occurring MOTUs. Figures below:

**^
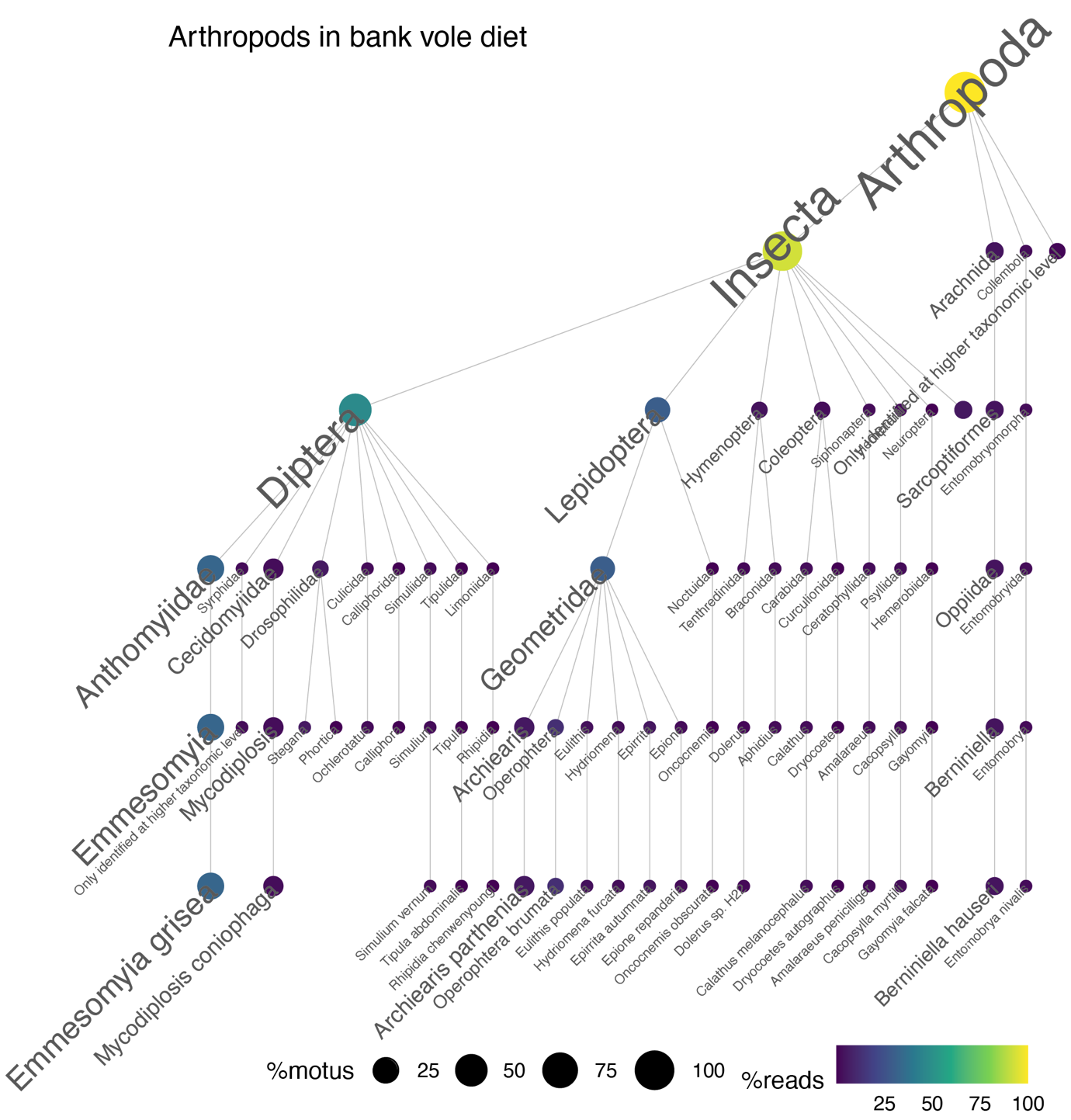

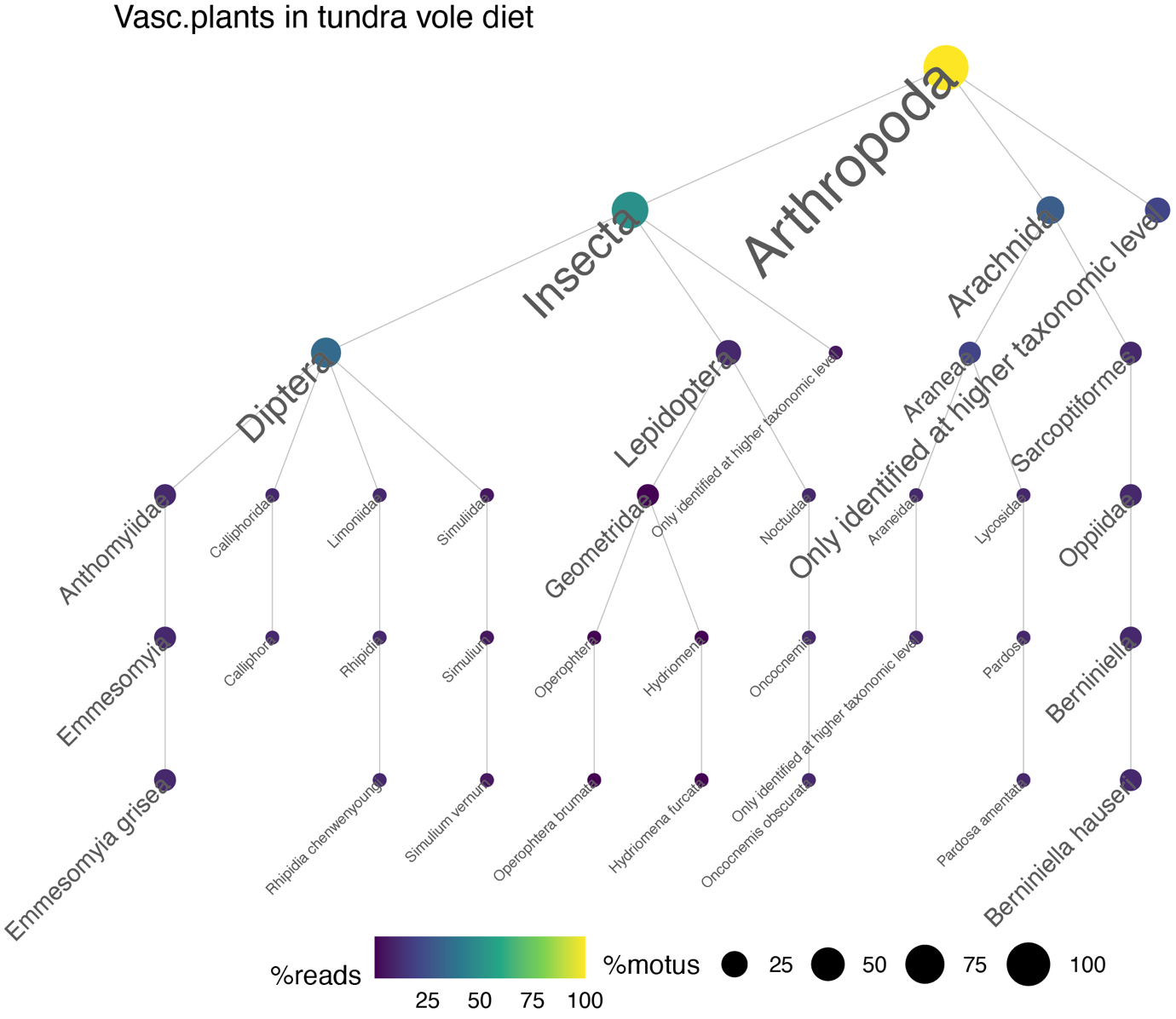
^**

**^
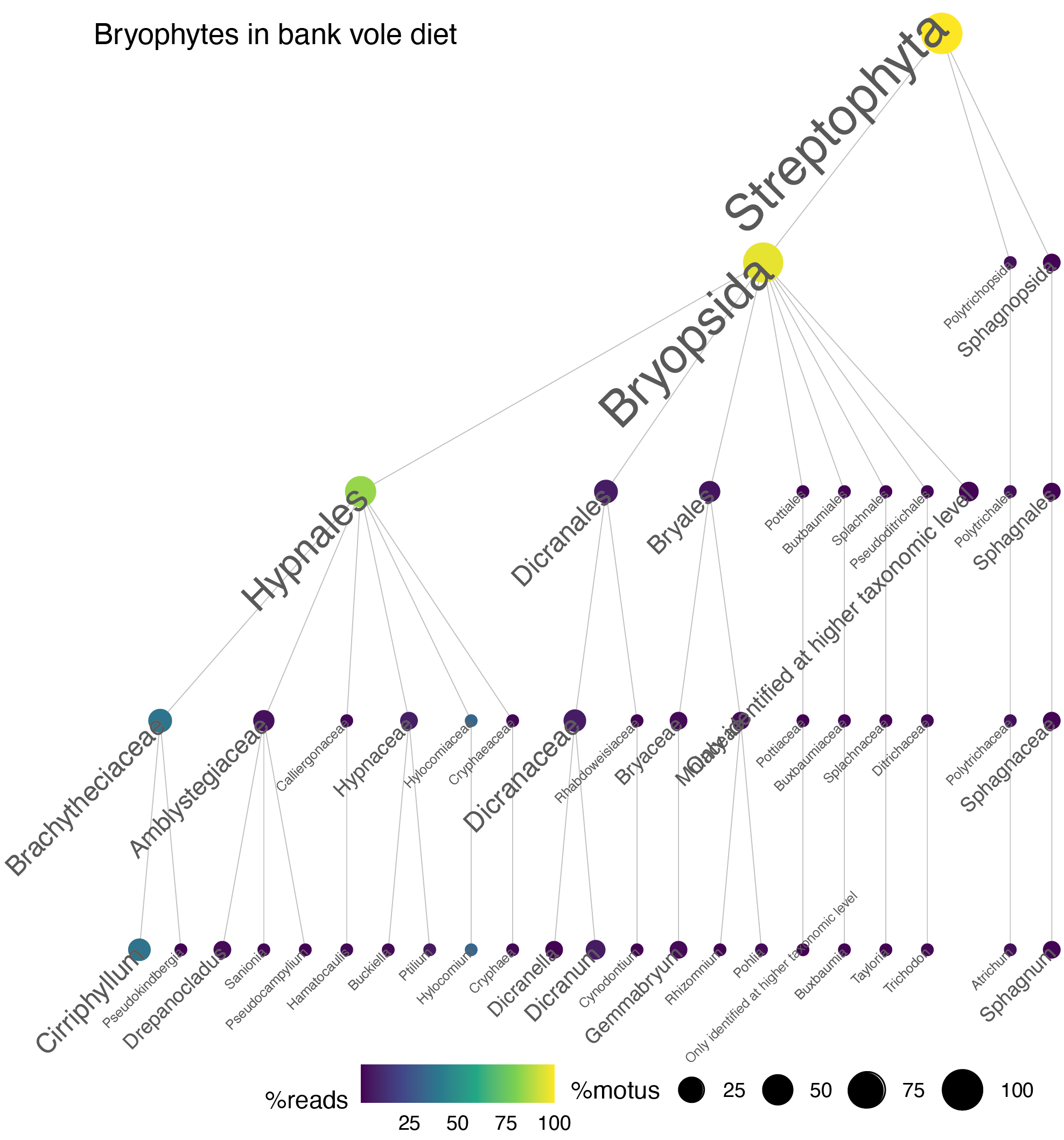

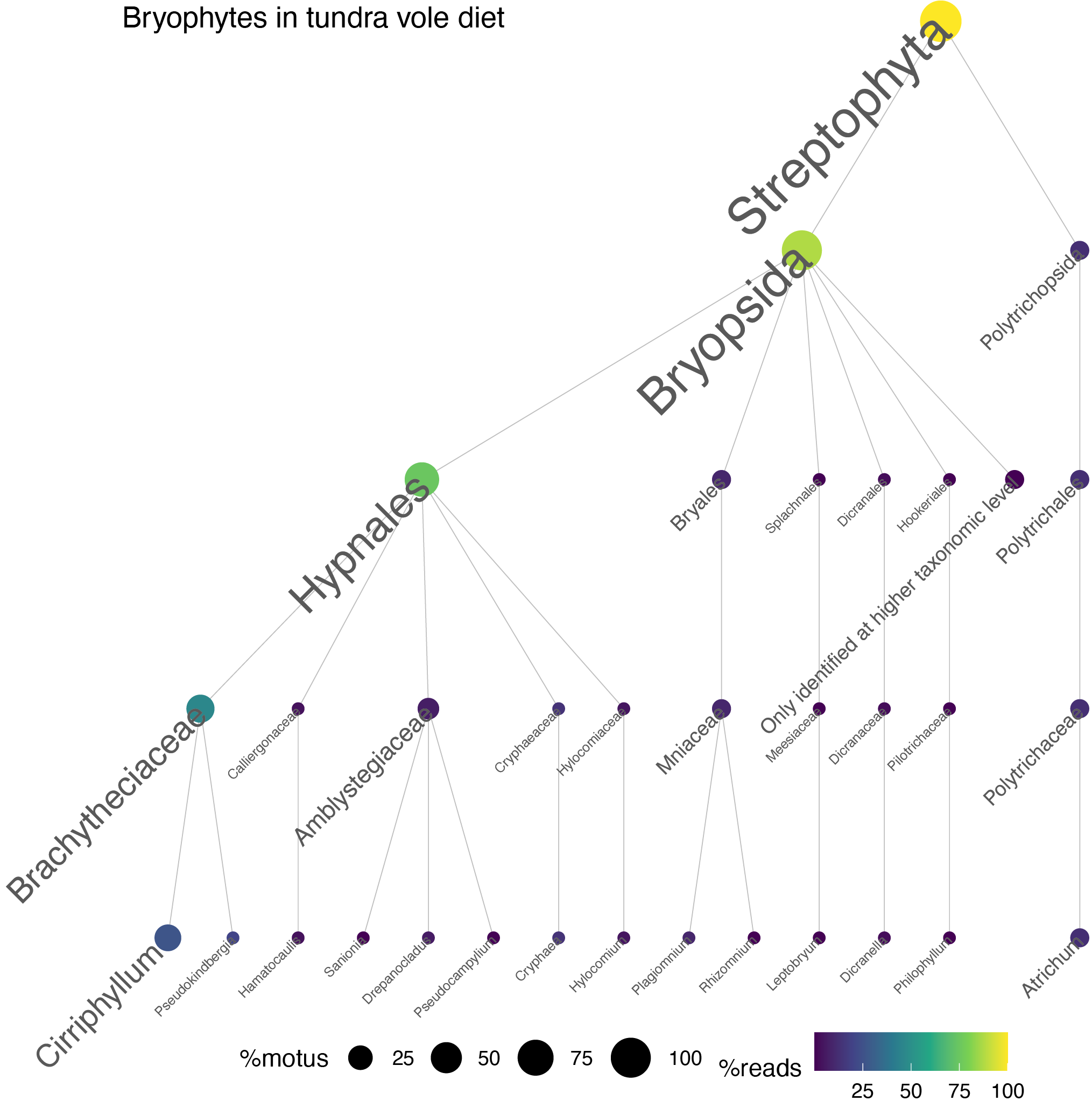

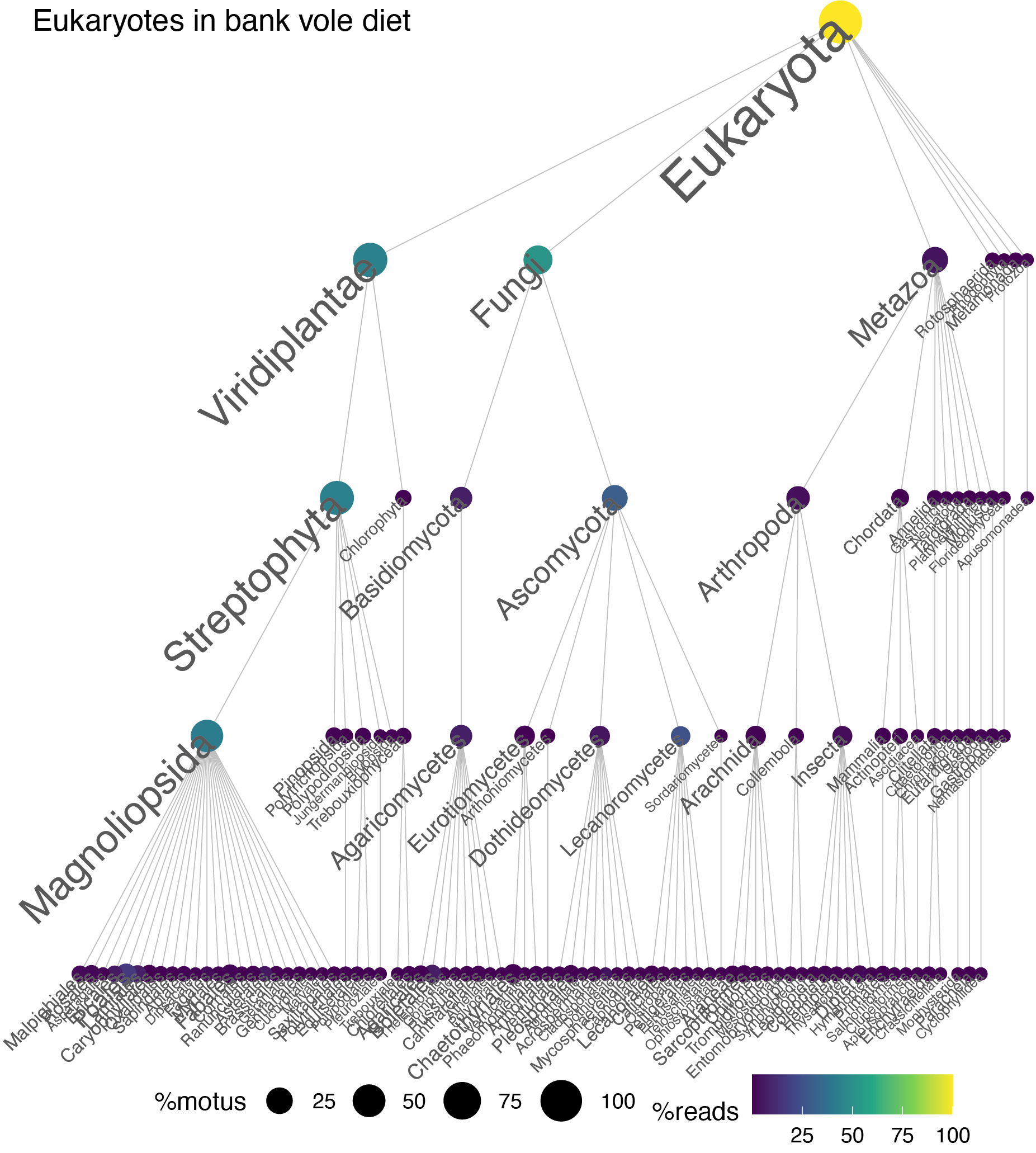

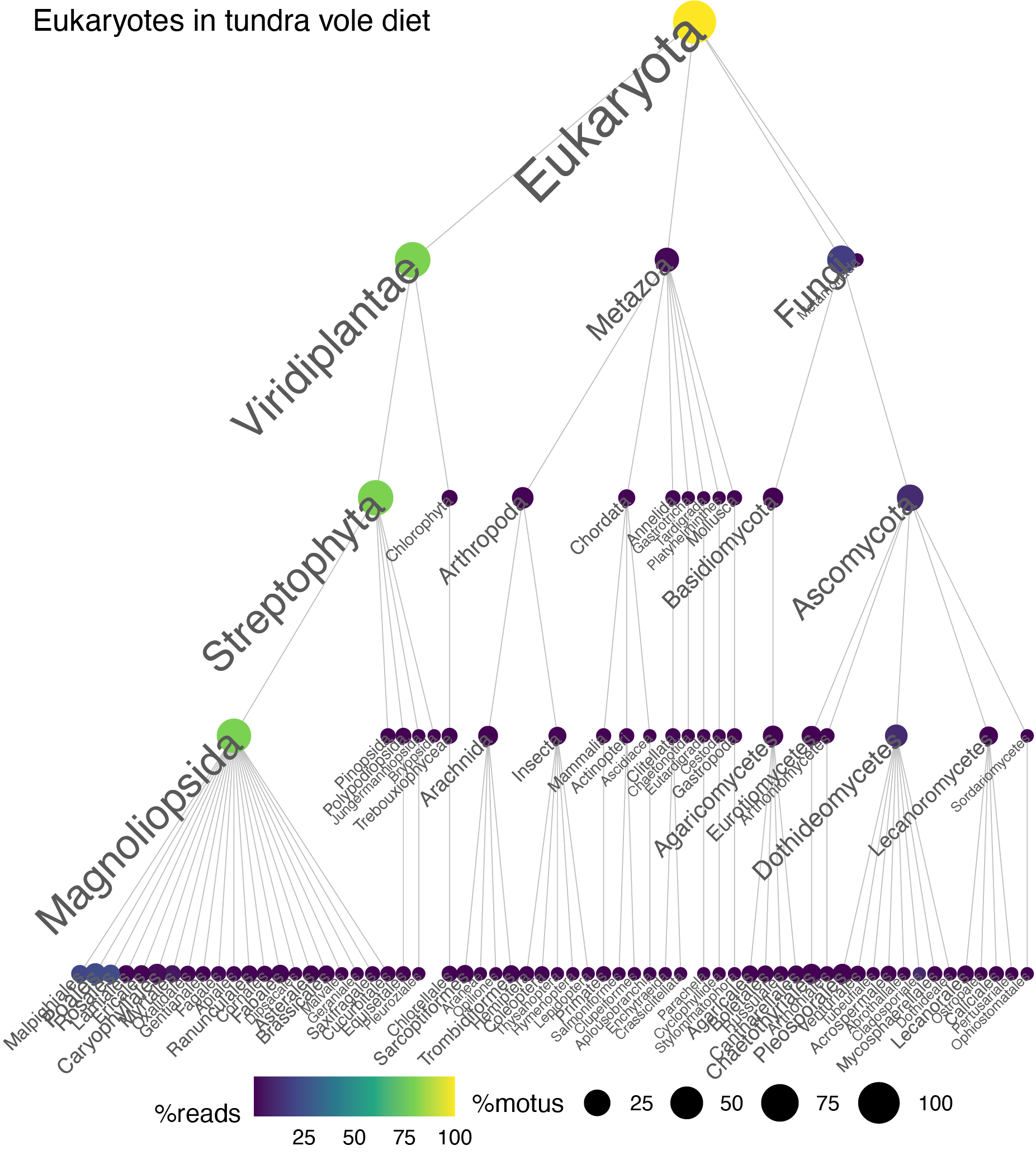

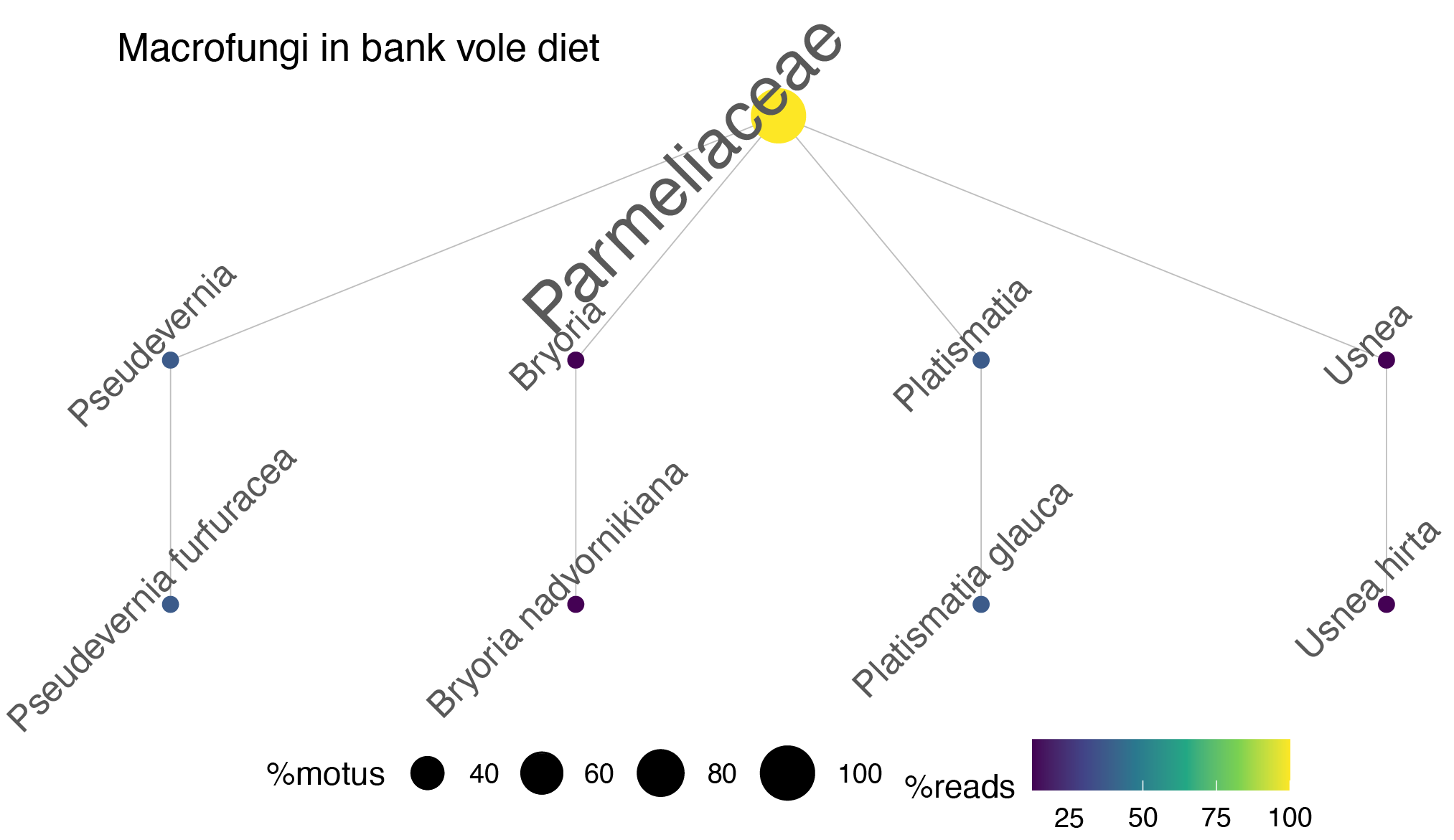

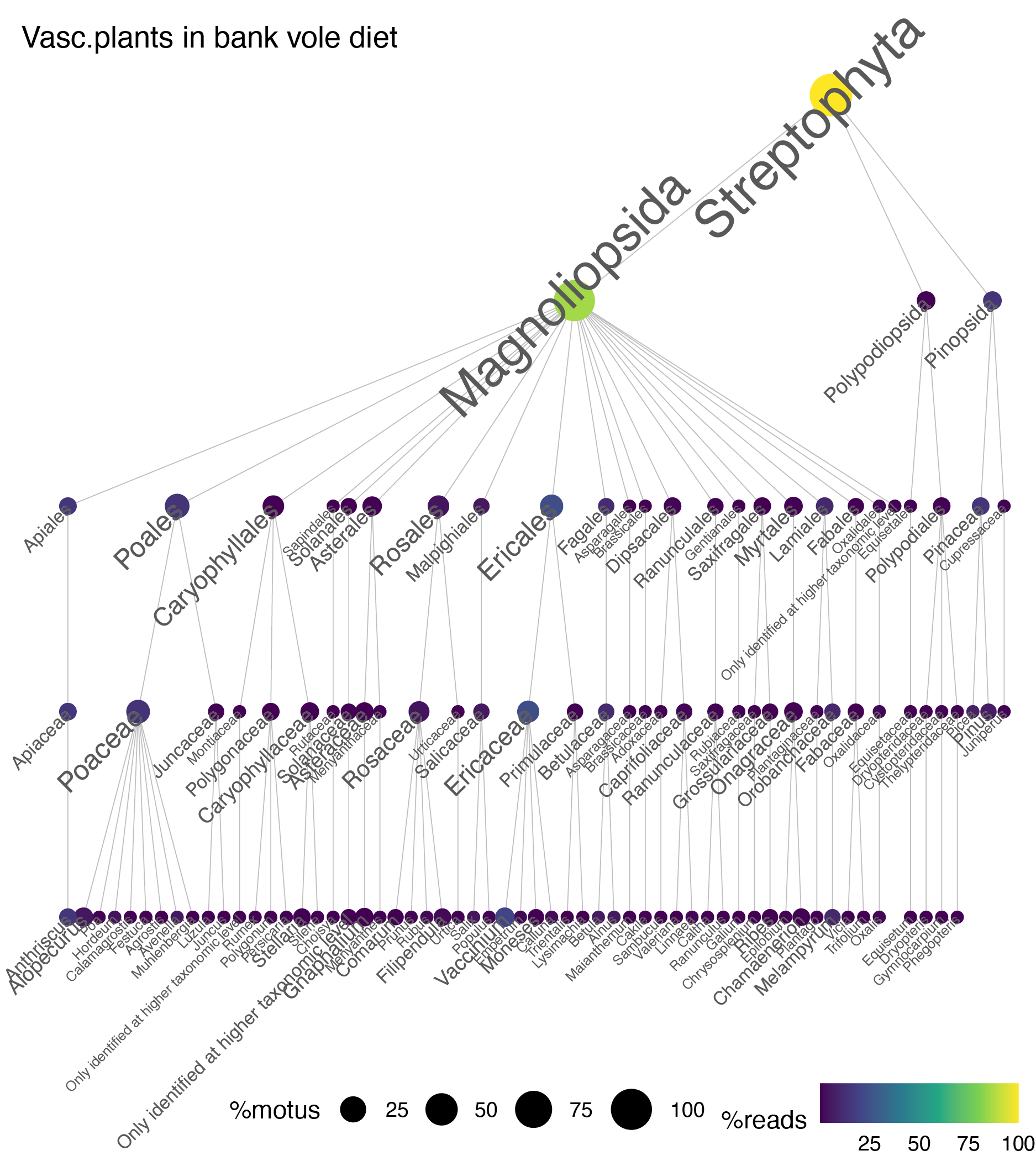

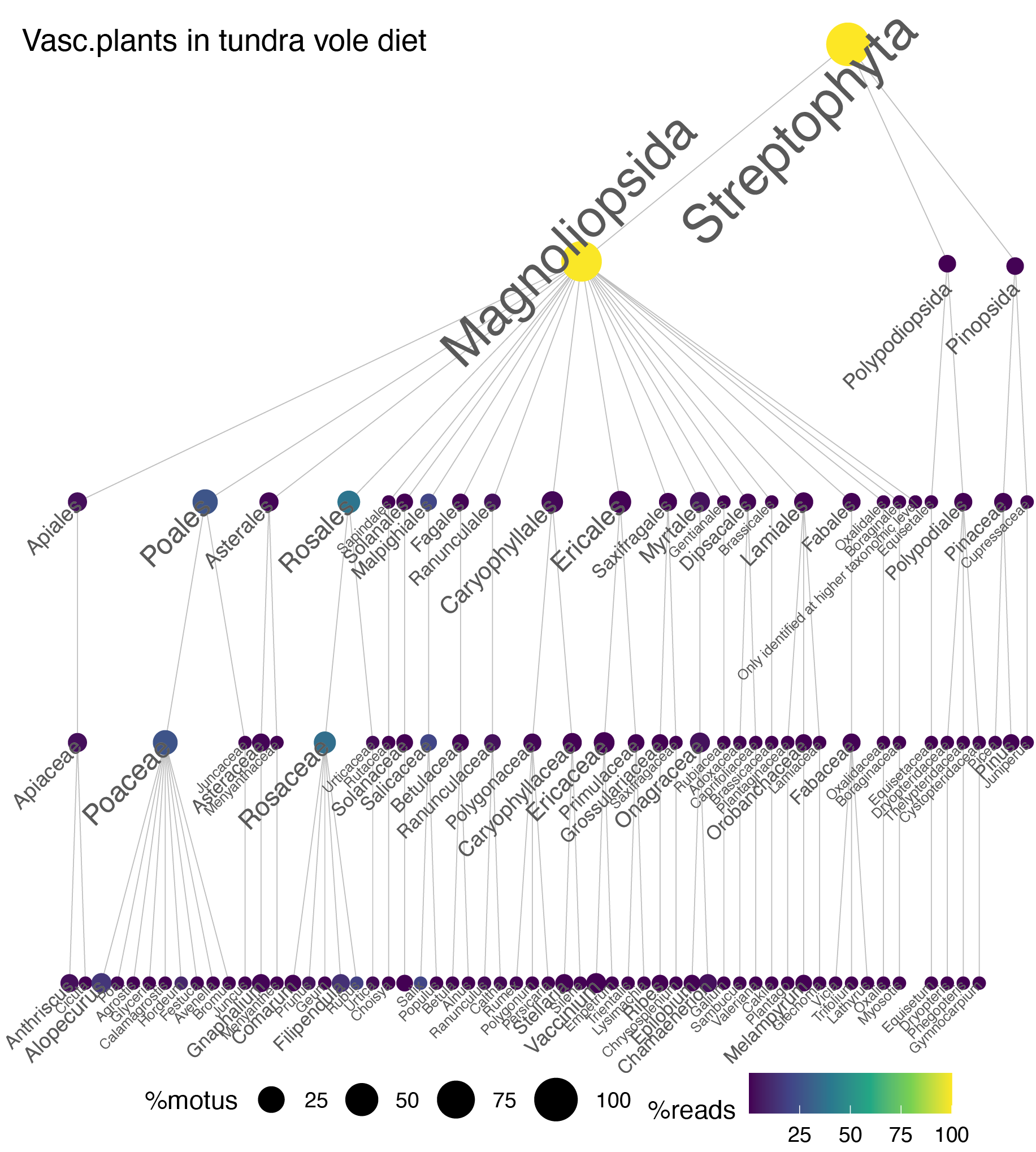
^**

**References**

Zinger L, Lionnet C, Benoiston AS, Donald J, Mercier C, Boyer F (2021) metabaR: An r package for the evaluation and improvement of DNA metabarcoding data quality. Methods in Ecology and Evolution 12:586-592. doi: 10.1111/2041-210x.13552
