# Supplementary material for: Is the diet cyclic phase‐dependent in boreal vole populations?
Source: Ecol Evol. 2024 Apr 17;14(4):e11227. doi: 10.1002/ece3.11227 (PMC11024456; doi:10.1002/ece3.11227)
Supplement: Supplementary file 6 — Appendix S6 [file ECE3-14-e11227-s006.docx]

**Online Resource 6 – Supplementary information on model outputs**

**Article name:** Is the diet cyclic phase-dependent in boreal vole populations?

**Journal name:** Unpublished manuscript

**Author names:** Magne Neby^1,2*^, Rolf A. Ims^3^, Stefaniya Kamenova^4,5^, Olivier Devineau^1^, Eeva M. Soininen^3^

^1^ Department of Applied Ecology, Inland Norway University of Applied Sciences, Koppang, Norway

^2^ Department of Agricultural Sciences, Inland Norway University of Applied Sciences, Hamar, Norway

^3^ Department of Arctic and Marine Biology, UiT – the Arctic University of Norway, Tromsø, Norway

^4^Centre for Ecological and Evolutionary Synthesis, Department of Biosciences, University of Oslo, 0316 Oslo, Norway

^5^Faculty of Environmental Sciences and Natural Resource Management, Norwegian University of Life Sciences, 1432 Ås, Norway

***Corresponding author:**

Magne Neby

Department of Agricultural Sciences, Inland Norway University of Applied Sciences, Høyvangvegen 40, 2322 Ridabu, Norway.

Email address: [magne.neby@inn.no](mailto:magne.neby@inn.no)

**Table ESM6.1.** The models found that 19.6 % and 7 % of the variation (Adjusted R^2^) with the Principal Component Analysis was explained by explanatory variables in the redundancy analysis (RDA) in the bank and tundra vole diets, respectively. PERMANOVA found significant differences in the diet composition (response variables) among all explanatory variables / groupings, in bank voles (B) and tundra voles (C). Here, (using adonis2 function in ade4 R package), terms were tested sequentially, but both order of the variables give p<0.05. Number of permutations were 999.

| A) |  |  |  |  |  |  |  |  |  |  |
| --- | --- | --- | --- | --- | --- | --- | --- | --- | --- | --- |
| **Population** | **N** | **Adj. R^2^** | **Eigenvalue** | | **Eigenvalue** | | **Prop. Explained** | | **Cumaltive prop.** | |
|  |  |  | **PC1** | **PC2** | **RDA1** | **RDA2** | **RDA1** | **RDA2** | **RDA1** | **RDA2** |
| *Myodes glareolus* | 127 | 20% | 0.16 | 0.08 | 0.07 | 0.01 | 0.65 | 0.10 | 0.65 | 0.75 |
| *Microtus oeconomus* | 66 | 7% | 0.08 | 0.06 | 0.02 | 0.01 | 0.90 | 0.10 | 0.90 | 0.99 |

| B) |  |  |  |  |  |
| --- | --- | --- | --- | --- | --- |
|  | **Df** | **SumOfSqs** | **R^2^** | **F** | **Pr(>F)** |
| phase:season | 3 | 3.6028 | 0.13352 | 6.5861 | 0.001 |
| grid | 6 | 2.283 | 0.08258 | 2.0368 | 0.001 |
| Residual | 116 | 21.1517 | 0.78390 |  |  |
| Total | 125 | 26.9828 | 1.00000 |  |  |
| C) |  |  |  |  |  |
|  | **Df** | **SumOfSqs** | **R^2^** | **F** | **Pr(>F)** |
| phase | 1 | 0.5346 | 0.05298 | 3.6961 | 0.002 |
| season | 1 | 0.1539 | 0.01525 | 1.0640 | 0.404 |
| Residual | 65 | 9.4024 | 0.93177 |  |  |
| Total | 67 | 10.0909 | 1.00000 |  |  |

| **Table ESM6.2.** Marginal Contrasts Analysis on glmm models with Relative Reads Abundance as response variable, and population cycle phase, season and plant functional group as additive explanatory variables, and sampling grid as random factor. None of the contrasts *within* the same plant functional group was significant on the alpha 0.05 level. | | | | | | | | |
| --- | --- | --- | --- | --- | --- | --- | --- | --- |
| **A) Tundra vole data** | |  |  |  |  |  |  |  |
| **G-H & RRA** |  |  |  |  |  |  |  |  |
| **Level1** | **Level2** | **Difference** | **CI_low** | **CI_high** | **SE** | **df** | **t** | **p** |
| crashLow summer forb | crashLow winter forb | -0.08 | -0.566 | 0.407 | 0.142 | 191 | -0.561 | 1 |
| crashLow summer forb | increasePeak winter forb | -0.177 | -0.563 | 0.209 | 0.113 | 191 | -1.571 | 0.917 |
| crashLow summer graminoid | crashLow winter graminoid | -0.08 | -0.566 | 0.407 | 0.142 | 191 | -0.561 | 1 |
| crashLow summer graminoid | increasePeak winter graminoid | -0.177 | -0.563 | 0.209 | 0.113 | 191 | -1.571 | 0.917 |
| crashLow summer shrub | crashLow winter shrub | -0.08 | -0.566 | 0.407 | 0.142 | 191 | -0.561 | 1 |
| crashLow summer shrub | increasePeak winter shrub | -0.177 | -0.563 | 0.209 | 0.113 | 191 | -1.571 | 0.917 |
| increasePeak summer forb | crashLow summer forb | 0.097 | -0.366 | 0.561 | 0.135 | 191 | 0.719 | 1 |
| increasePeak summer forb | crashLow winter forb | 0.018 | -0.851 | 0.886 | 0.254 | 191 | 0.069 | 1 |
| increasePeak summer forb | increasePeak winter forb | -0.08 | -0.566 | 0.407 | 0.142 | 191 | -0.561 | 1 |
| increasePeak summer graminoid | crashLow summer graminoid | 0.097 | -0.366 | 0.561 | 0.135 | 191 | 0.719 | 1 |
| increasePeak summer graminoid | crashLow winter graminoid | 0.018 | -0.851 | 0.886 | 0.254 | 191 | 0.069 | 1 |
| increasePeak summer graminoid | increasePeak winter graminoid | -0.08 | -0.566 | 0.407 | 0.142 | 191 | -0.561 | 1 |
| increasePeak summer shrub | crashLow summer shrub | 0.097 | -0.366 | 0.561 | 0.135 | 191 | 0.719 | 1 |
| increasePeak summer shrub | crashLow winter shrub | 0.018 | -0.851 | 0.886 | 0.254 | 191 | 0.069 | 1 |
| increasePeak summer shrub | increasePeak winter shrub | -0.08 | -0.566 | 0.407 | 0.142 | 191 | -0.561 | 1 |
| increasePeak winter forb | crashLow winter forb | 0.097 | -0.366 | 0.561 | 0.135 | 191 | 0.719 | 1 |
| increasePeak winter graminoid | crashLow winter graminoid | 0.097 | -0.366 | 0.561 | 0.135 | 191 | 0.719 | 1 |
| increasePeak winter shrub | crashLow winter shrub | 0.097 | -0.366 | 0.561 | 0.135 | 191 | 0.719 | 1 |
| **EUKA & RRA** |  |  |  |  |  |  |  |  |
| **Level1** | **Level2** | **Difference** | **CI_low** | **CI_high** | **SE** | **df** | **t** | **p** |
| crashLow summer forb | crashLow winter forb | -0.011 | -0.134 | 0.113 | 0.036 | 188 | -0.299 | 1 |
| crashLow summer forb | increasePeak winter forb | -0.031 | -0.129 | 0.066 | 0.028 | 188 | -1.098 | 0.994 |
| crashLow summer graminoid | crashLow winter graminoid | -0.011 | -0.134 | 0.113 | 0.036 | 188 | -0.299 | 1 |
| crashLow summer graminoid | increasePeak winter graminoid | -0.031 | -0.129 | 0.066 | 0.028 | 188 | -1.098 | 0.994 |
| crashLow summer shrub | crashLow winter shrub | -0.011 | -0.134 | 0.113 | 0.036 | 188 | -0.299 | 1 |
| crashLow summer shrub | increasePeak winter shrub | -0.031 | -0.129 | 0.066 | 0.028 | 188 | -1.098 | 0.994 |
| increasePeak summer forb | crashLow summer forb | 0.02 | -0.096 | 0.137 | 0.034 | 188 | 0.598 | 1 |
| increasePeak summer forb | crashLow winter forb | 0.01 | -0.21 | 0.23 | 0.064 | 188 | 0.149 | 1 |
| increasePeak summer forb | increasePeak winter forb | -0.011 | -0.134 | 0.113 | 0.036 | 188 | -0.299 | 1 |
| increasePeak summer graminoid | crashLow summer graminoid | 0.02 | -0.096 | 0.137 | 0.034 | 188 | 0.598 | 1 |
| increasePeak summer graminoid | crashLow winter graminoid | 0.01 | -0.21 | 0.23 | 0.064 | 188 | 0.149 | 1 |
| increasePeak summer graminoid | increasePeak winter graminoid | -0.011 | -0.134 | 0.113 | 0.036 | 188 | -0.299 | 1 |
| increasePeak summer shrub | crashLow summer shrub | 0.02 | -0.096 | 0.137 | 0.034 | 188 | 0.598 | 1 |
| increasePeak summer shrub | crashLow winter shrub | 0.01 | -0.21 | 0.23 | 0.064 | 188 | 0.149 | 1 |
| increasePeak summer shrub | increasePeak winter shrub | -0.011 | -0.134 | 0.113 | 0.036 | 188 | -0.299 | 1 |
| increasePeak winter forb | crashLow winter forb | 0.02 | -0.096 | 0.137 | 0.034 | 188 | 0.598 | 1 |
| increasePeak winter graminoid | crashLow winter graminoid | 0.02 | -0.096 | 0.137 | 0.034 | 188 | 0.598 | 1 |
| increasePeak winter shrub | crashLow winter shrub | 0.02 | -0.096 | 0.137 | 0.034 | 188 | 0.598 | 1 |
|  |  |  |  |  |  |  |  |  |
| **B) Bank vole data** |  |  |  |  |  |  |  |  |
| **G-H & RRA** |  |  |  |  |  |  |  |  |
| **Level1** | **Level2** | **Difference** | **CI_low** | **CI_high** | **SE** | **df** | **t** | **p** |
| crashLow winter forb | crashLow summer forb | 0.003 | -0.262 | 0.267 | 0.078 | 370 | 0.035 | 1 |
| crashLow winter forb | increasePeak summer forb | 0.009 | -0.422 | 0.44 | 0.127 | 370 | 0.07 | 1 |
| crashLow winter graminoid | crashLow summer graminoid | 0.003 | -0.262 | 0.267 | 0.078 | 370 | 0.035 | 1 |
| crashLow winter graminoid | increasePeak summer graminoid | 0.009 | -0.422 | 0.44 | 0.127 | 370 | 0.07 | 1 |
| crashLow winter shrub | crashLow summer shrub | 0.003 | -0.262 | 0.267 | 0.078 | 370 | 0.035 | 1 |
| crashLow winter shrub | increasePeak summer shrub | 0.009 | -0.422 | 0.44 | 0.127 | 370 | 0.07 | 1 |
| increasePeak summer forb | crashLow summer forb | -0.006 | -0.294 | 0.281 | 0.085 | 370 | -0.072 | 1 |
| increasePeak summer graminoid | crashLow summer graminoid | -0.006 | -0.294 | 0.281 | 0.085 | 370 | -0.072 | 1 |
| increasePeak summer shrub | crashLow summer shrub | -0.006 | -0.294 | 0.281 | 0.085 | 370 | -0.072 | 1 |
| increasePeak winter forb | crashLow summer forb | -0.003 | -0.349 | 0.342 | 0.102 | 370 | -0.033 | 1 |
| increasePeak winter forb | crashLow winter forb | -0.006 | -0.294 | 0.281 | 0.085 | 370 | -0.072 | 1 |
| increasePeak winter forb | increasePeak summer forb | 0.003 | -0.262 | 0.267 | 0.078 | 370 | 0.035 | 1 |
| increasePeak winter graminoid | crashLow summer graminoid | -0.003 | -0.349 | 0.342 | 0.102 | 370 | -0.033 | 1 |
| increasePeak winter graminoid | crashLow winter graminoid | -0.006 | -0.294 | 0.281 | 0.085 | 370 | -0.072 | 1 |
| increasePeak winter graminoid | increasePeak summer graminoid | 0.003 | -0.262 | 0.267 | 0.078 | 370 | 0.035 | 1 |
| increasePeak winter shrub | crashLow summer shrub | -0.003 | -0.349 | 0.342 | 0.102 | 370 | -0.033 | 1 |
| increasePeak winter shrub | crashLow winter shrub | -0.006 | -0.294 | 0.281 | 0.085 | 370 | -0.072 | 1 |
| increasePeak winter shrub | increasePeak summer shrub | 0.003 | -0.262 | 0.267 | 0.078 | 370 | 0.035 | 1 |
| **EUKA & RRA** |  |  |  |  |  |  |  |  |
| **Level1** | **Level2** | **Difference** | **CI_low** | **CI_high** | **SE** | **df** | **t** | **p** |
| crashLow winter forb | crashLow summer forb | -0.21 | -0.461 | 0.041 | 0.074 | 343 | -2.839 | 0.168 |
| crashLow winter forb | increasePeak summer forb | -0.188 | -0.602 | 0.226 | 0.122 | 343 | -1.546 | 0.927 |
| crashLow winter graminoid | crashLow summer graminoid | -0.21 | -0.461 | 0.041 | 0.074 | 343 | -2.839 | 0.168 |
| crashLow winter graminoid | increasePeak summer graminoid | -0.188 | -0.602 | 0.226 | 0.122 | 343 | -1.546 | 0.927 |
| crashLow winter shrub | crashLow summer shrub | -0.21 | -0.461 | 0.041 | 0.074 | 343 | -2.839 | 0.168 |
| crashLow winter shrub | increasePeak summer shrub | -0.188 | -0.602 | 0.226 | 0.122 | 343 | -1.546 | 0.927 |
| increasePeak summer forb | crashLow summer forb | -0.021 | -0.293 | 0.25 | 0.08 | 343 | -0.267 | 1 |
| increasePeak summer graminoid | crashLow summer graminoid | -0.021 | -0.293 | 0.25 | 0.08 | 343 | -0.267 | 1 |
| increasePeak summer shrub | crashLow summer shrub | -0.021 | -0.293 | 0.25 | 0.08 | 343 | -0.267 | 1 |
| increasePeak winter forb | crashLow summer forb | -0.231 | -0.55 | 0.088 | 0.094 | 343 | -2.458 | 0.37 |
| increasePeak winter forb | crashLow winter forb | -0.021 | -0.293 | 0.25 | 0.08 | 343 | -0.267 | 1 |
| increasePeak winter forb | increasePeak summer forb | -0.21 | -0.461 | 0.041 | 0.074 | 343 | -2.839 | 0.168 |
| increasePeak winter graminoid | crashLow summer graminoid | -0.231 | -0.55 | 0.088 | 0.094 | 343 | -2.458 | 0.37 |
| increasePeak winter graminoid | crashLow winter graminoid | -0.021 | -0.293 | 0.25 | 0.08 | 343 | -0.267 | 1 |
| increasePeak winter graminoid | increasePeak summer graminoid | -0.21 | -0.461 | 0.041 | 0.074 | 343 | -2.839 | 0.168 |
| increasePeak winter shrub | crashLow summer shrub | -0.231 | -0.55 | 0.088 | 0.094 | 343 | -2.458 | 0.37 |
| increasePeak winter shrub | crashLow winter shrub | -0.021 | -0.293 | 0.25 | 0.08 | 343 | -0.267 | 1 |
| increasePeak winter shrub | increasePeak summer shrub | -0.21 | -0.461 | 0.041 | 0.074 | 343 | -2.839 | 0.168 |
